# Hgc1 Independence of Biofilm Hyphae in Candida albicans

**DOI:** 10.1128/mbio.03498-22

**Published:** 2023-02-13

**Authors:** Anupam Sharma, Norma V. Solis, Manning Y. Huang, Frederick Lanni, Scott G. Filler, Aaron P. Mitchell

**Affiliations:** a Department of Microbiology, University of Georgia, Athens, Georgia, USA; b Lundquist Institute for Biomedical Innovation at Harbor-UCLA Medical Center, Torrance, California, USA; c Department of Biological Sciences, Carnegie Mellon University, Pittsburgh, Pennsylvania, USA; d David Geffen School of Medicine at UCLA, Los Angeles, California, USA; Yonsei University

**Keywords:** biofilm, *Candida*, hyphae, regulation, virulence

## Abstract

Biofilm and hypha formation are central to virulence of the fungal pathogen Candida albicans. The G1 cyclin gene *HGC1* is required for hypha formation under diverse *in vitro* and *in vivo* growth conditions. Hgc1 is required for disseminated infection and is a linchpin in the argument that hyphal morphogenesis itself is required for pathogenicity. We report here that *HGC1* is dispensable for hypha formation during biofilm formation both *in vitro*, under strong inducing conditions, and *in vivo*, in a mouse oropharyngeal candidiasis model. These findings are validated with two or more C. albicans isolates. Systematic screening of overexpressed cyclin genes indicates that *CCN1* and *CLN3* can compensate partially for Hgc1 function during biofilm growth. This conclusion is also supported by the severity of the *hgc1*Δ/Δ *ccn1*Δ/Δ double mutant biofilm defect. Our results suggest that hypha formation in biofilm is accomplished by combined action of multiple cyclins, not solely by Hgc1.

## INTRODUCTION

The opportunistic fungal pathogen Candida albicans is the primary cause of invasive candidiasis, with mortality rates of 30 to 40% (1). It colonizes and infects mucosal surfaces, organs, and tissues. In addition, C. albicans can form multicellular biofilm communities on tissues and implanted medical devices, evading host immunity and antifungal treatment (2).

C. albicans can grow in both yeast and filamentous forms. Yeast are unicellular ovoid-shaped cells that grow by budding. Filamentous forms include both pseudohyphae and hyphae. Pseudohyphae are ellipsoid cells with constrictions at the septation site, and hyphae are long parallel-sided cells with no such constriction (3). Hyphae grow by tip extension and emerge from a yeast cell upon induction as a highly polarized germ tube (3). Adherence of hyphae to human epithelial and endothelial cells enables invasion of host tissue (4, 5). Hypha formation is significant because it is required for both biofilm formation and virulence (5).

The hypha-associated gene *HGC1* (hyphal specific G1 cyclin 1) encodes a cyclin with strongest similarity to the G1 family of cyclins and is indispensable for hyphal growth under many environmental conditions (6). Hgc1 associates with cyclin-dependent kinase Cdc28 to direct continuous growth at the hyphal tip (6) through phosphorylation of diverse effector proteins (7, 8). Like many genes required for hypha formation (9, 10), *HGC1* is required for biofilm formation under some conditions (11, 12). Importantly, *HGC1* is required for virulence in a disseminated infection model (6, 11). The virulence defect of an *hgc1*Δ/Δ mutant is a key element in the hypothesis that hyphal growth itself is required for virulence (7, 13).

There are two additional G1 cyclins in C. albicans, Cln3 and Ccn1. Cln3 is essential for yeast cell propagation (14–16) and functions as a negative regulator of hyphal morphogenesis (15–17). Specifically, repression of *CLN3* expression under yeast growth conditions causes production of hyphal filaments (15, 16). Ccn1 is not essential for growth, but it is required for normal hyphal morphogenesis under some growth conditions, including solid medium or nutritionally poor medium (18, 19). While *ccn1*Δ/Δ mutants initiate hypha formation as efficiently as the respective wild-type (WT) strain, they revert to yeast-form growth after prolonged incubation, when the wild-type strain continues to produce hyphae (20). Therefore, Ccn1 is considered to promote maintenance of hyphal growth but not initiation (19, 20).

Hyphae support virulence of C. albicans in many ways (9). Prominent among these is biofilm formation, which depends upon hypha formation in almost all contexts investigated (9, 10). The determinants of biofilm formation have been characterized extensively (9, 10). Biofilm formation depends upon Hgc1 in a well-established assay system with Spider medium (12). However, imaging was not used in that study to determine whether the *hgc1*Δ/Δ mutant grew as hyphae in the nominal biofilm that was produced. In fact, the authors pointed out that the precise role of Hgc1 in biofilm formation was uncertain (12).

In recent studies, we have sought to define the genetic determinants of biofilm formation among multiple C. albicans clinical isolates (21, 22). We have found that biofilm determinants vary considerably from strain to strain (21, 22). Our studies thus far have examined the regulatory network that controls expression of hypha-associated genes like *HGC1.* Because Hgc1 is a downstream target of the network, and because it has defined mechanistic roles in hyphal morphogenesis (7, 13), we anticipated that it would be required for biofilm formation in all C. albicans strains. The simple rationale for our study led us to a result that we consider astonishing: an *hgc1*Δ/Δ mutation does not abolish hypha formation under biofilm growth conditions. We document the finding in several C. albicans strains and in two distinct biofilm models. Follow-up analysis argues that G1 cyclins Ccn1 and Cln3 contribute to hyphal development and biofilm growth and that they may overcome the absence of Hgc1 to promote hypha formation in the context of biofilm growth.

## RESULTS

### Natural variation in the impact of Hgc1 on pathogenicity traits.

We constructed *hgc1Δ/Δ* mutants in five clinical isolates (21, 23, 24): SC5314 (clade 1), P76067 (clade 2), P57055 (clade 3), GC75 (clade 4), and 19F (clade 1). SC5314, P76067, and GC75 undergo filamentation strongly under several growth conditions, while P57055 and 19F undergo filamentation weakly (see below and references 21 and 23).

**FIG 1:**
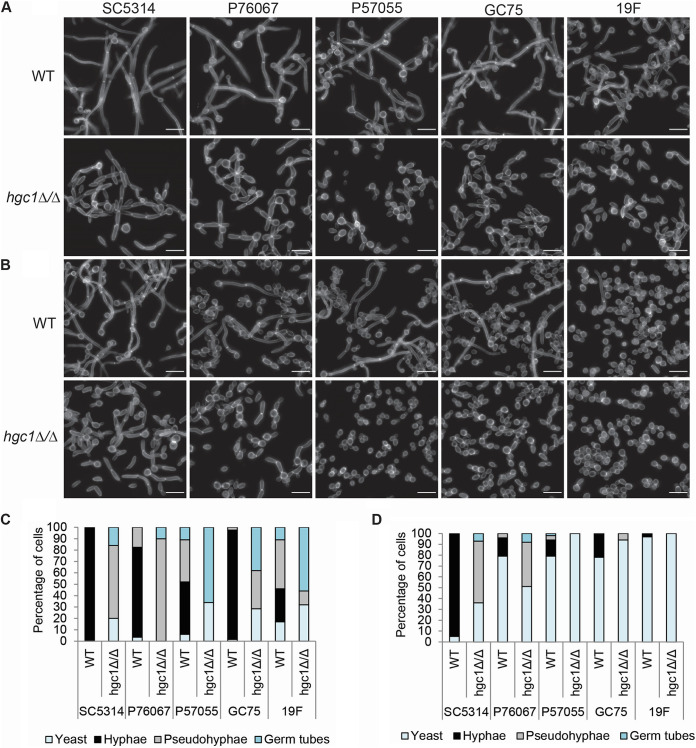
Phenotype of *hgc1*Δ/Δ mutants in diverse strain backgrounds. Cell morphology of wild-type and *hgc1*Δ/Δ strains of each strain background grown in RPMI plus 10% serum (A and C) or Spider medium (B and D) at 37°C for 4 h. The white scale bars in each panel are 16 μm in length. Cells were classified as hyphae when the filaments were narrow (<2 μm) and had parallel sides. Cells were classified as pseudohyphae when the width was >2 μm, there was a constriction, and a septum at the neck of the mother cell-daughter compartment, and the sides were not parallel. Cells were classified as short germ tubes which swelled to form a less-polarized structure. A minimum of five fields and 100 cells were classified for each strain.

Wild-type and mutant strains were assayed for hypha formation under planktonic (nonbiofilm, free-living) growth conditions. In RPMI-plus-serum medium at 37°C, the wild-type strains all produced hyphae, though the extent of hypha formation varied (Fig. 1A and C). SC5314, P76067, and GC75 *hgc1*Δ/Δ mutants yielded predominantly pseudohyphae, and P57055 and 19F *hgc1*Δ/Δ mutants yielded mainly short germ tubes and yeast cells (Fig. 1A and C). Reconstituted derivatives of the *hgc1*Δ/Δ mutants, in which two copies of *HGC1* from SC5314 were introduced at the native locus, regained the ability to form hyphae comparable to the respective wild-type strain (see Fig. S1 in the supplemental material). In Spider medium at 37°C, all wild-type strains except SC5314 produced hyphae less efficiently than in RPMI plus serum (Fig. 1B and D). SC5314 and P76067 *hgc1*Δ/Δ mutants yielded pseudohyphae and yeast cells, and P57055, GC75, and 19F *hgc1*Δ/Δ mutants yielded mainly yeast cells (Fig. 1B and D). These observations indicate that overall filamentation of each mutant strain varies in proportion to filamentation of the respective wild-type strain. In every case, though, the *hgc1*Δ/Δ mutation prevented detectable formation of hyphae. These results support the current understanding of Hgc1 function and extend that understanding to multiple C. albicans isolates.

10.1128/mbio.03498-22.1 FIG S1 Filamentation assay of reconstituted strains. The *HGC1* allele from SC5314 was reconstituted in the mutants of all clinical isolates. The reconstituted strains were grown in RPMI plus 10% serum at 37°C for 4 h alongside wild-type and *hgc1*Δ/Δ mutant strains in the corresponding clinical isolate backgrounds. Fixed cells were stained with calcofluor white and imaged using fluorescence microscopy. The white scale bars in each panel are 16 μm in length. Download FIG S1, PDF file, 0.2 MB. Copyright © 2023 Sharma et al. 2023 Sharma et al. https://creativecommons.org/licenses/by/4.0/ This content is distributed under the terms of the Creative Commons Attribution 4.0 International license.

**FIG 2:**
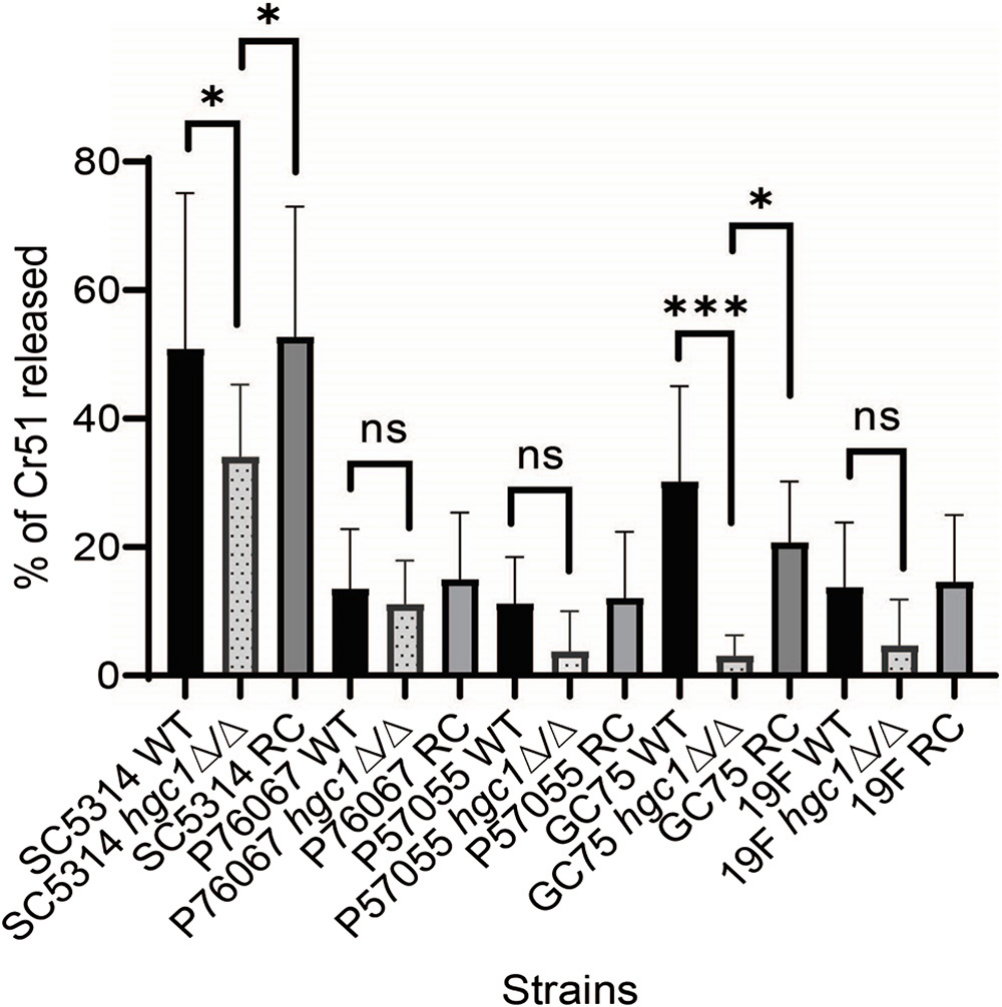
Impact of *HGC1* deletion on virulence traits. Human endothelial cells were incubated with the indicated strains of C. albicans for 3 h, after which the extent of host cell damage was determined using a ^51^Cr release assay. Results are the mean from three independent experiments, each performed in triplicate. Error bars denote standard deviation. Data were analyzed using Bonferroni’s multicomparison test (ns, *P* > 0.05; *, *P* < 0.05; ***, *P* < 0.001).

Hgc1 is required for pathogenicity in systemic mouse and zebrafish infection models (6, 25). For many C. albicans mutants, defects in systemic infection capacity correlate with defects in endothelial cell damage capacity (26–28). Therefore, we tested the five wild-type, *hgc1*Δ/Δ, and complemented strains for ability to damage human endothelial cells. Strains SC5314 and GC75 inflicted more damage than the other wild-type strains (Fig. 2); the corresponding *hgc1*Δ/Δ mutants exhibited reduced damage ability, which was restored by complementation (Fig. 2). The P76067, P57055, and 19F *hgc1*Δ/Δ mutants trended toward a reduction in damage compared to the wild-type strains, but the differences were not statistically significant. These results indicate that *hgc1*Δ/Δ mutations can cause endothelial cell damage defects; detection of this phenotype may depend upon strong damage ability of the wild-type strain. Because hyphal defects are associated with endothelial cell damage defects (4, 27), these results are consistent with current understanding of Hgc1 function.

### Dispensability of Hgc1 for biofilm formation.

**FIG 3:**
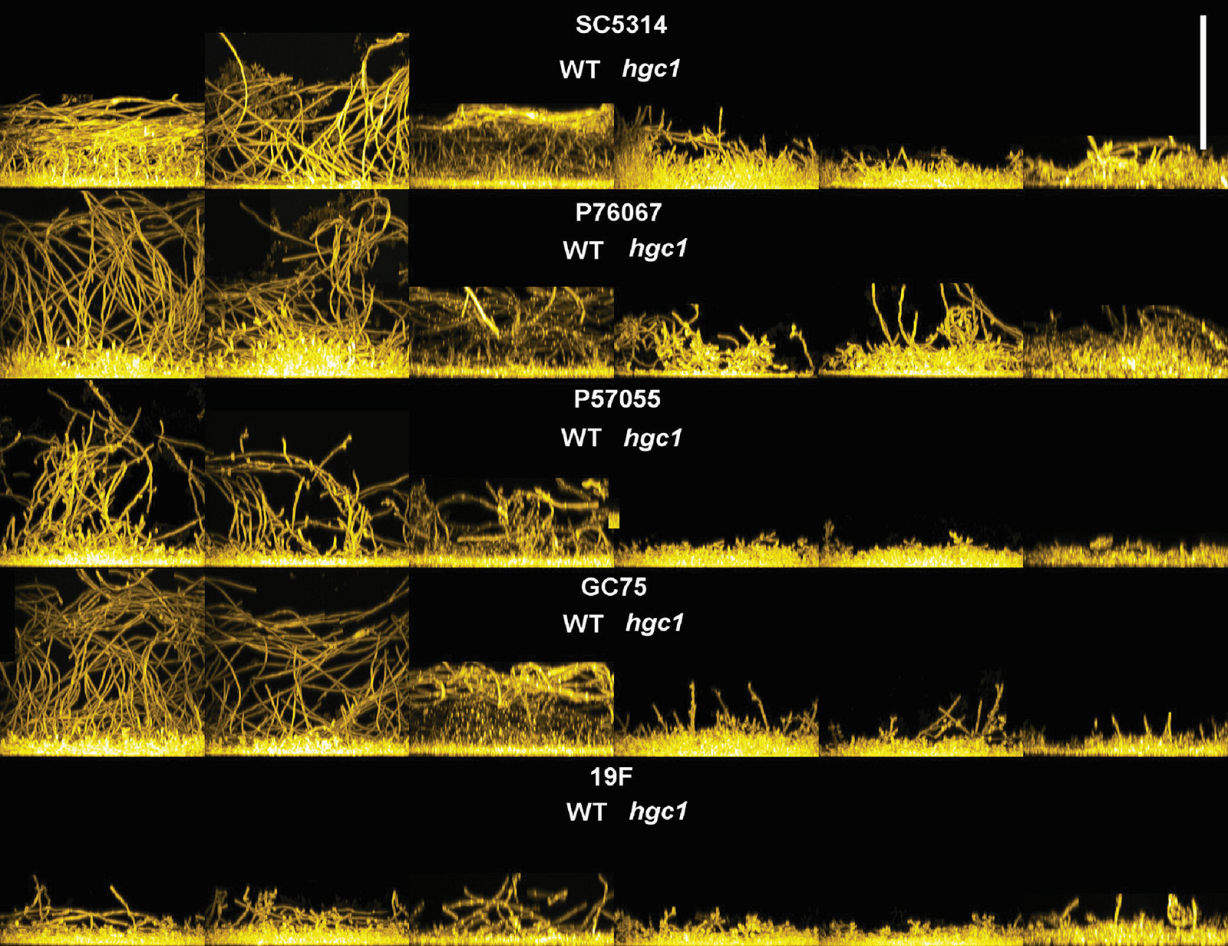
Effect of *HGC1* deletion on C. albicans biofilm formation. Side view projections of biofilms. Wild-type and mutant strains in each strain background were assayed for biofilm formation under *in vitro* conditions. Strains were grown under in RPMI plus 10% serum at 37°C for 24 h (96-well plate). Fixed biofilms were stained using calcofluor white and then visualized by confocal microscopy. Side projections of a biofilm of each strain (in triplicate), WT (left) and respective *hgc1*Δ/Δ mutant (right), are shown. The white scale bar represents 100 μm in length.

In prior studies, Hgc1 was found to be required for biofilm formation (12, 29). We sought to assay the impact of *hgc1*Δ/Δ mutations on biofilm formation in multiple strain backgrounds. We used RPMI-plus-serum medium at 37°C, a condition that induces hyphae strongly and promotes biofilm formation by all wild-type strains in this study (Fig. 3). Under our conditions, SC5314, P76067, and GC75 produced biofilm depth of ~60 to 90 μm, while P57055 and 19F produced biofilm depth of 25 to 60 μm. In all strain backgrounds, *hgc1*Δ/Δ mutants produced biofilm, though depth was slightly reduced compared to the respective wild-type strain (Fig. 3). Quantitative XTT [2,3-bis-(2-methoxy-4-nitro-5-sulfophenyl)-2H-tetrazolium-5-carboxanilide salt] dye reduction assays confirmed that *hgc1*Δ/Δ mutants had reduced biofilm biomass (Fig. S2). These results indicate that Hgc1 is required for normal biofilm biomass in all five strains examined. However, under our conditions, *hgc1*Δ/Δ mutants are clearly capable of biofilm formation.

10.1128/mbio.03498-22.2 FIG S2 XTT reduction assay. The effect of *hgc1*Δ/Δ mutation on the biofilm growth across five clinical isolates was measured in RPMI plus 10% serum using the XTT assay. Error bars indicate standard deviations from 3 biological replicates assayed. Data were analyzed using Sidak’s multicomparison test (****, *P* < 0.0001). Download FIG S2, PDF file, 0.3 MB. Copyright © 2023 Sharma et al. 2023 Sharma et al. https://creativecommons.org/licenses/by/4.0/ This content is distributed under the terms of the Creative Commons Attribution 4.0 International license.

**FIG 4:**
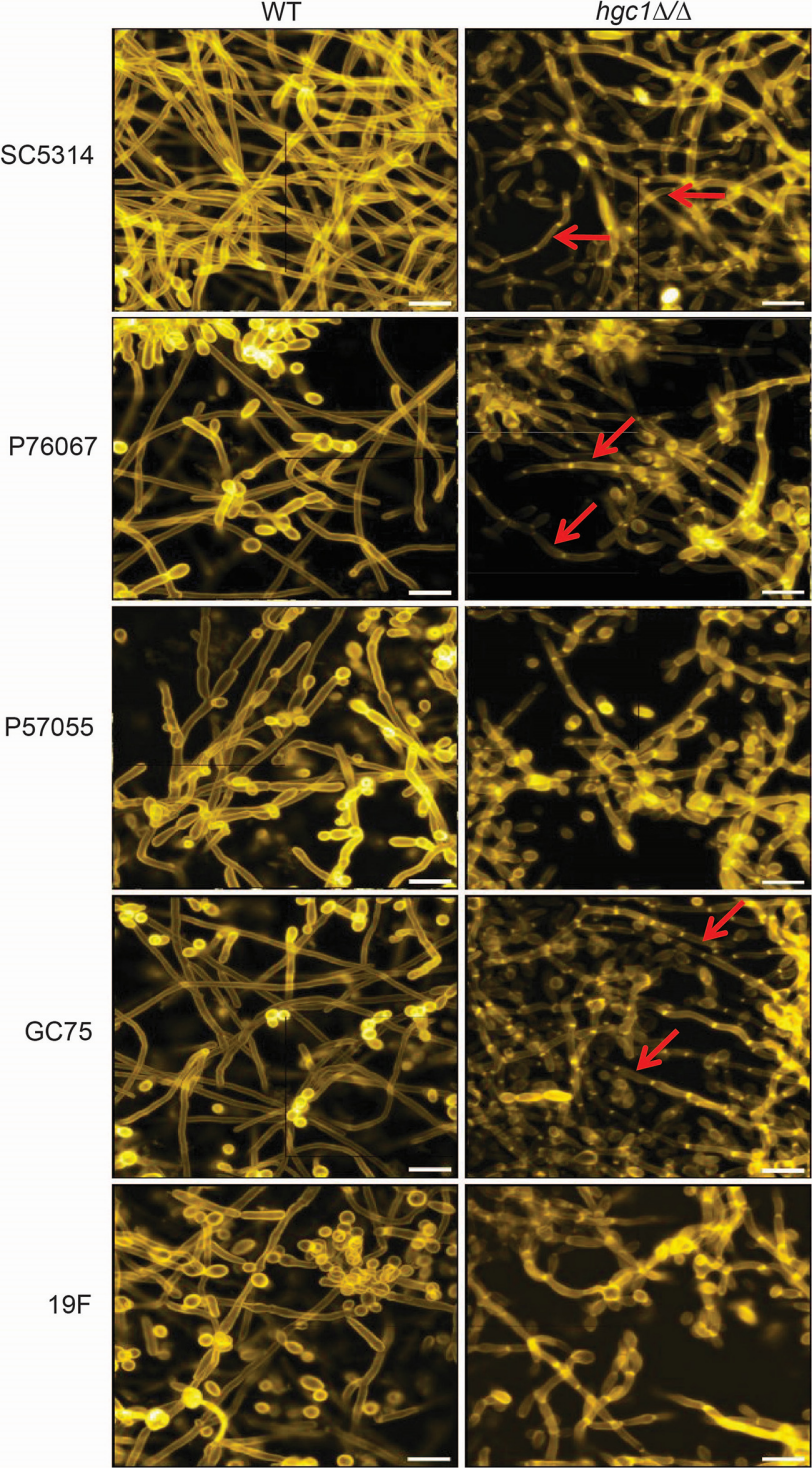
Apical view projections of biofilms. Apical view projections of indicated strains were obtained (biofilm sets from Fig. 3) using maximum-intensity Z-projection of ~50 planes at an 0.85-μm step size. Biofilm images were magnified to improve cell type visualization. Red arrows point toward the hyphae.

The *hgc1*Δ/Δ mutant biofilms had a striking structural feature. Long, highly polarized hyphae were present in mutant biofilms in the SC5314, P76067, and GC75 strain backgrounds (Fig. 4). In contrast, filaments in the *hgc1*Δ/Δ mutant biofilms of P57055 and 19F, which were less numerous, had evident constrictions like pseudohyphae. The ability of *hgc1*Δ/Δ mutants to produce long hyphae conflicts with current understanding of Hgc1 function (7, 13, 17).

**FIG 5:**
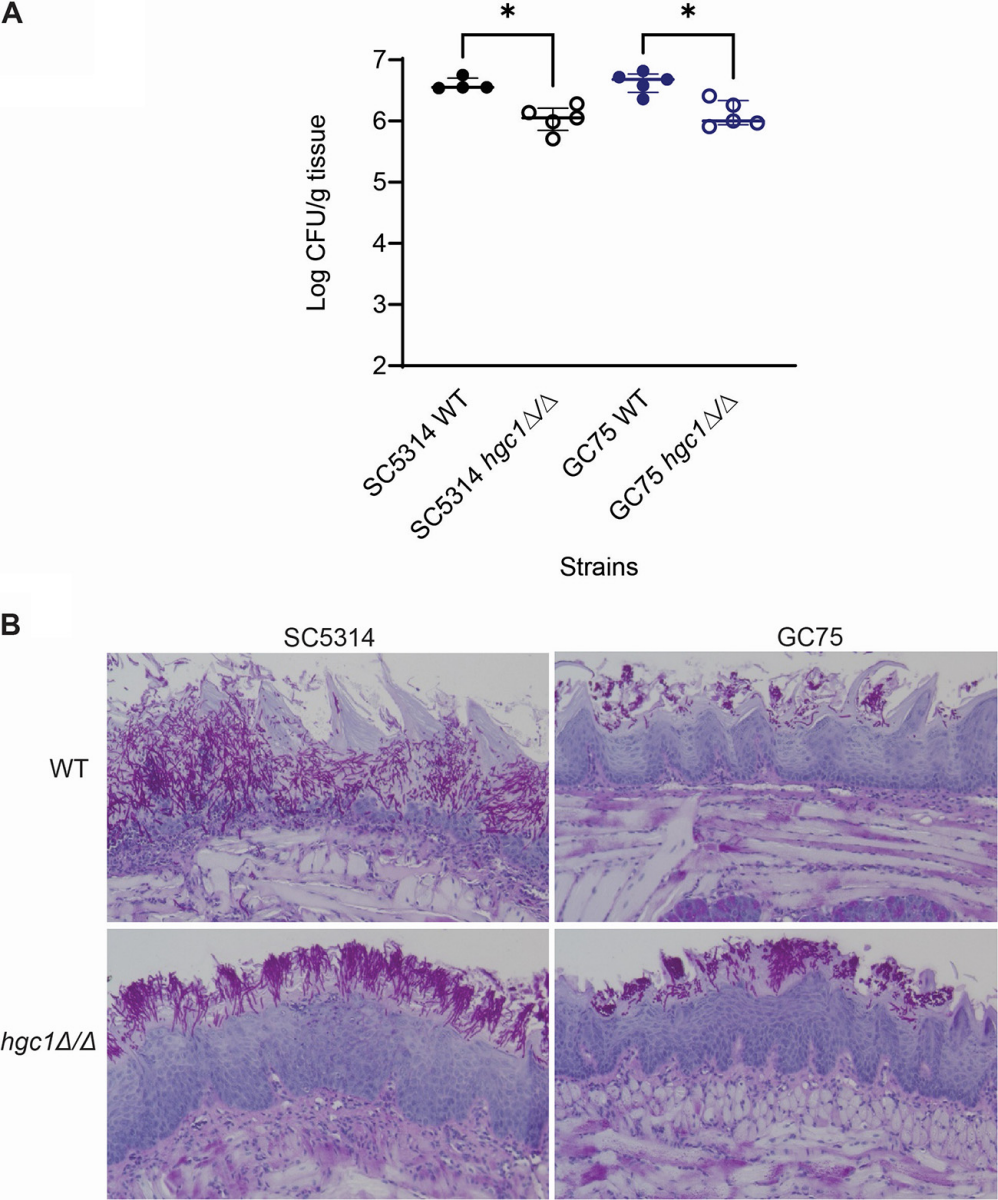
*hgc1*Δ/Δ mutation attenuates virulence during oropharyngeal candidiasis. (A) Oral fungal burden of immunosuppressed mice after 5 days of infection with the indicated strains of C. albicans. Results are medians ± interquartile ranges from 5 mice per strain/phenotype. Data were analyzed using Dunn’s multicomparison test (*, *P* < 0.05). (B) Tongue histopathology after 5 days of infection.

Given that *hgc1*Δ/Δ mutants produce hyphae during biofilm growth *in vitro*, we sought to extend our studies to a biofilm-based infection model, the oral mucosal infection model (30–32). We used SC5314, the type strain, as well as GC75, which is highly virulent in a mouse systemic infection model (24). Immunosuppressed mice were inoculated orally, and fungal burdens were assayed at 5 days postinfection. The two wild-type strains produced similar fungal burdens (Fig. 5A); both *hgc1*Δ/Δ mutants had slightly reduced fungal burdens (Fig. 5A). Histopathological examination of infected tongues revealed that hyphae of wild-type SC5314 invaded the entire tongue epithelium. The corresponding *hgc1*Δ/Δ mutant also formed hyphae, though invasion was more superficial (Fig. 5B). The wild-type GC75 produced short hyphae and caused only superficial invasion in this model (Fig. 5B). The corresponding *hgc1*Δ/Δ mutant behaved similarly (Fig. 5B). We conclude that Hgc1 is required for maximal virulence during oral infection in both strains. However, Hgc1 is not required for hypha formation in the oral environment. This observation conflicts with the current understanding of the Hgc1 function (7, 13, 17). In addition, the finding that *hgc1*Δ/Δ mutants of two different genetic backgrounds form hyphae in this infection model indicates that hypha formation in the absence of Hgc1 is not unique to one strain background.

### Contribution of G1 cyclins Ccn1 and Cln3 to biofilm growth.

**FIG 6:**
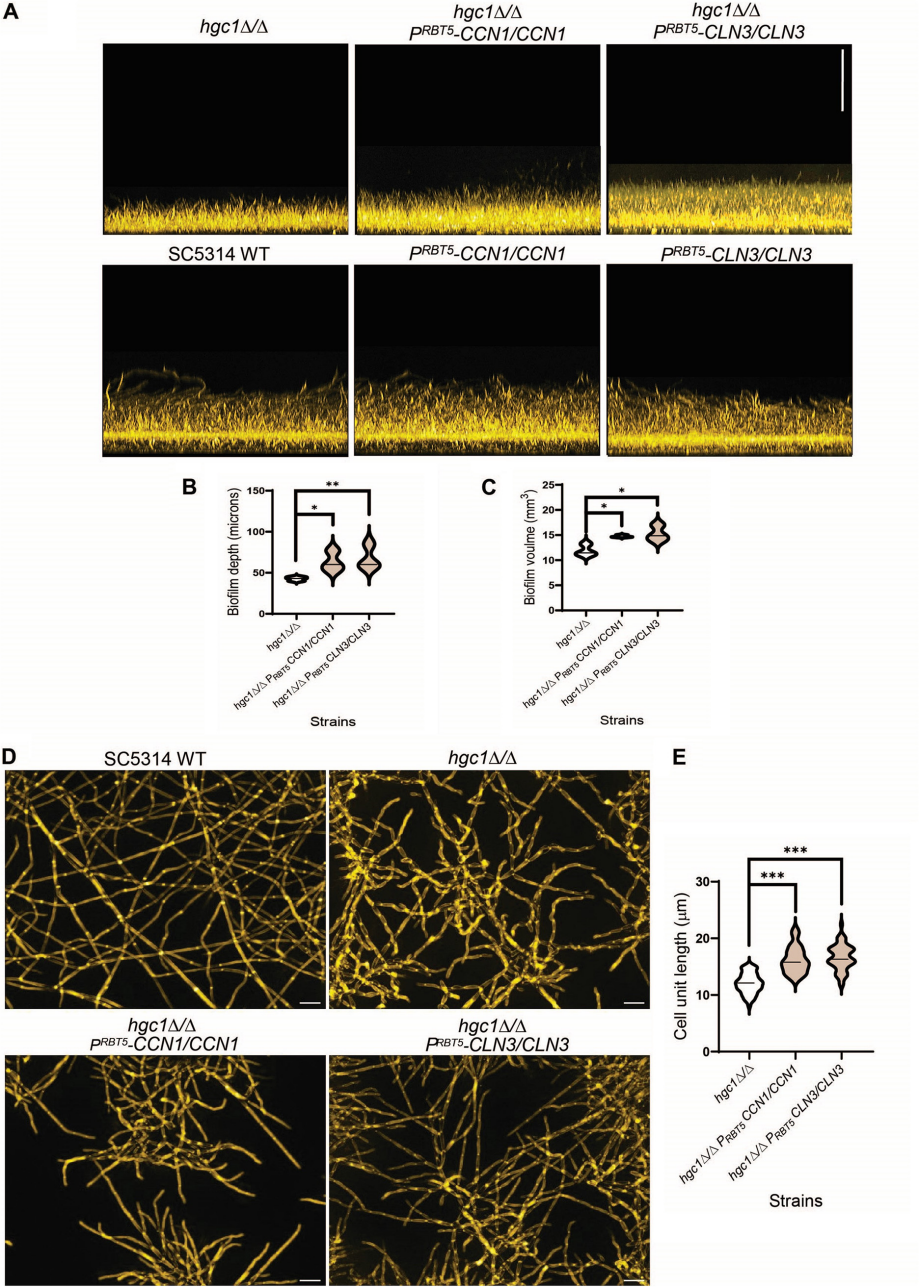
G1 cyclin genes *CCN1* and *CLN3* partially rescue the biofilm growth defect of SC5314 *hgc1*Δ/Δ mutant. (A) Biofilm side view projections. Wild-type SC5314 and its derivative strains were assayed for biofilm formation under *in vitro* conditions. All strains were grown in RPMI at 37°C for 24 h (96-well plate). Fixed biofilms were stained using calcofluor white and imaged using a Keyence BZ-X800E fluorescence microscope. Representative side view images are shown, and the vertical scale bar represents 100 μm in length. (B and C) Violin plots showing the biofilm depth (in micrometers) and volume (in cubic millimeters) distributions of the indicated strains, respectively. The biofilm depth is the measure of the height of the top surface of the biofilm, whereas biofilm volume is the measure of the total number of pixels of biofilm stain in three-dimensional space. Biofilm depth and volume were measured with the software ImageJ. The significant differences were calculated between the pair of means using Sidak’s multicomparison test: *, *P* < 0.05; **, *P* < 0.01; ns, *P* > 0.05. (D) Apical view projections from the biofilms of the indicated strains were obtained using maximum intensity Z-projection of ~20 planes at an 0.45-μm step size. White scale bars in each panel are 100 μm in length. (E) Violin plots showing the distribution of cell unit lengths of the indicated strains. Cell unit lengths were quantified with ImageJ, with a minimum of five fields and 50 cells for each strain background. The significant differences were calculated between the pair of means using Sidak’s multicomparison test: ***, *P* < 0.001.

We hypothesized that the other G1 cyclin genes *CCN1* and *CLN3* may compensate for *HGC1* function under biofilm growth conditions. We first investigated cyclin gene function through overexpression in the SC5314 wild-type and *hgc1*Δ/Δ strains. To overexpress the cyclin genes, we constructed a panel of heterozygous strains, each with one allele of a cyclin gene fused to the *RBT5* promoter (33). The *RBT5* promoter is repressed under the iron-sufficient growth conditions used for routine growth (33), thus minimizing potential for overexpression-related growth defects. The promoter is induced when strains are grown under iron-limited conditions, such as RPMI medium, and is similar in strength to the *TDH3* promoter (33). In order to detect possible improvement of biofilm growth, we used RPMI medium without serum, in which *hgc1*Δ/Δ mutants produce more pseudohyphae than in RPMI plus serum. Wild-type SC5314 and its overexpression strains yielded biofilms of similar depth, though overexpression of *CCN1* and *CLN3* caused slightly increased biofilm volume (Fig. S4 and S6). Overexpression of *CCN1* and *CLN3* in the *hgc1*Δ/Δ mutant increased both biofilm depth and volume (Fig. 6A, B, and C). These observations argue that G1 cyclins Ccn1 and Cln3 can compensate for loss of Hgc1 function during biofilm growth. We extended this conclusion with cell unit length measurements (Fig. 6D). The *hgc1*Δ/Δ mutant grew predominantly as pseudohyphae with less polarized hyphal filaments in RPMI medium (Fig. 6D). Increased expression of either *CCN1* or *CLN3* in the *hgc1*Δ/Δ background significantly increased the cell unit length (Fig. 6D and E). The features of both biofilm and cell units indicate that the G1 cyclin genes *CCN1* and *CLN3* contribute to hyphal growth in the absence of Hgc1.

10.1128/mbio.03498-22.4 FIG S4 Overexpression of cyclin genes *PCL1* and *PCL7* induces hyphal growth during biofilm formation in SC5314 *hgc1*Δ/Δ mutant. (A and B) Biofilm side view projections. Wild-type SC5314 and its derivative strains were assayed for biofilm formation under *in vitro* conditions. All strains were grown in RPMI at 37°C for 24 h (96-well plate). Fixed biofilms were stained using calcofluor white and imaged using a Keyence BZ-X800E fluorescence microscope. Representative side view images are shown, and the vertical scale bar represents 100 μm in length. (C and D) Violin plots showing the biofilm depth (in micrometers) and volume (in cubic millimeters) distributions of the indicated strains, respectively. Biofilm depth and volume were measured with the software ImageJ. The significant differences were calculated between the pair of means using Sidak’s multicomparison test: ns, *P* > 0.05. (E) Apical view projections from the biofilms of the indicated strains were obtained using maximum-intensity Z-projection of ~20 planes at an 0.45-μm step size. White scale bars in each panel are 100 μm in length. (F) Violin plots showing the distribution of cell unit lengths of the indicated strains. Cell unit lengths were quantified with ImageJ, with a minimum of five fields and 50 cells for each strain background. The significant differences were calculated between the pair of means using Sidak’s multicomparison test: **, *P* < 0.01; ***, *P* < 0.001. Download FIG S4, PDF file, 0.4 MB. Copyright © 2023 Sharma et al. 2023 Sharma et al. https://creativecommons.org/licenses/by/4.0/ This content is distributed under the terms of the Creative Commons Attribution 4.0 International license.

10.1128/mbio.03498-22.5 FIG S5 Biofilm depth and volume of SC5314 wild-type strains overexpressing cyclin genes. Biofilm depth (in micrometers) and volume (in cubic millimeters) of all the mentioned strains grown in RPMI medium at 37°C for 24 h were measured with Image J. (A and B) Violin plots showing the biofilm depth (in micrometers) and volume (in cubic millimeters) distributions of the indicated strains, respectively. The significant differences were calculated between the pair of means using Sidak’s multicomparison test: ns, *P* > 0.05; *, *P* < 0.05; ***, *P* < 0.001. Download FIG S5, PDF file, 0.2 MB. Copyright © 2023 Sharma et al. 2023 Sharma et al. https://creativecommons.org/licenses/by/4.0/ This content is distributed under the terms of the Creative Commons Attribution 4.0 International license.

10.1128/mbio.03498-22.6 FIG S6 Biofilm depth and volume of strains overexpressing different cyclin genes. Biofilm depth (in micrometers) and volume (in cubic millimeters) of all the mentioned strains grown in RPMI medium at 37°C for 24 h were measured with Image J. The values represent the means from the triplicate biofilms of each strain background. Download FIG S6, PDF file, 0.2 MB. Copyright © 2023 Sharma et al. 2023 Sharma et al. https://creativecommons.org/licenses/by/4.0/ This content is distributed under the terms of the Creative Commons Attribution 4.0 International license.

### Genetic interaction of *HGC1* and *CCN1* during biofilm growth.

**FIG 7:**
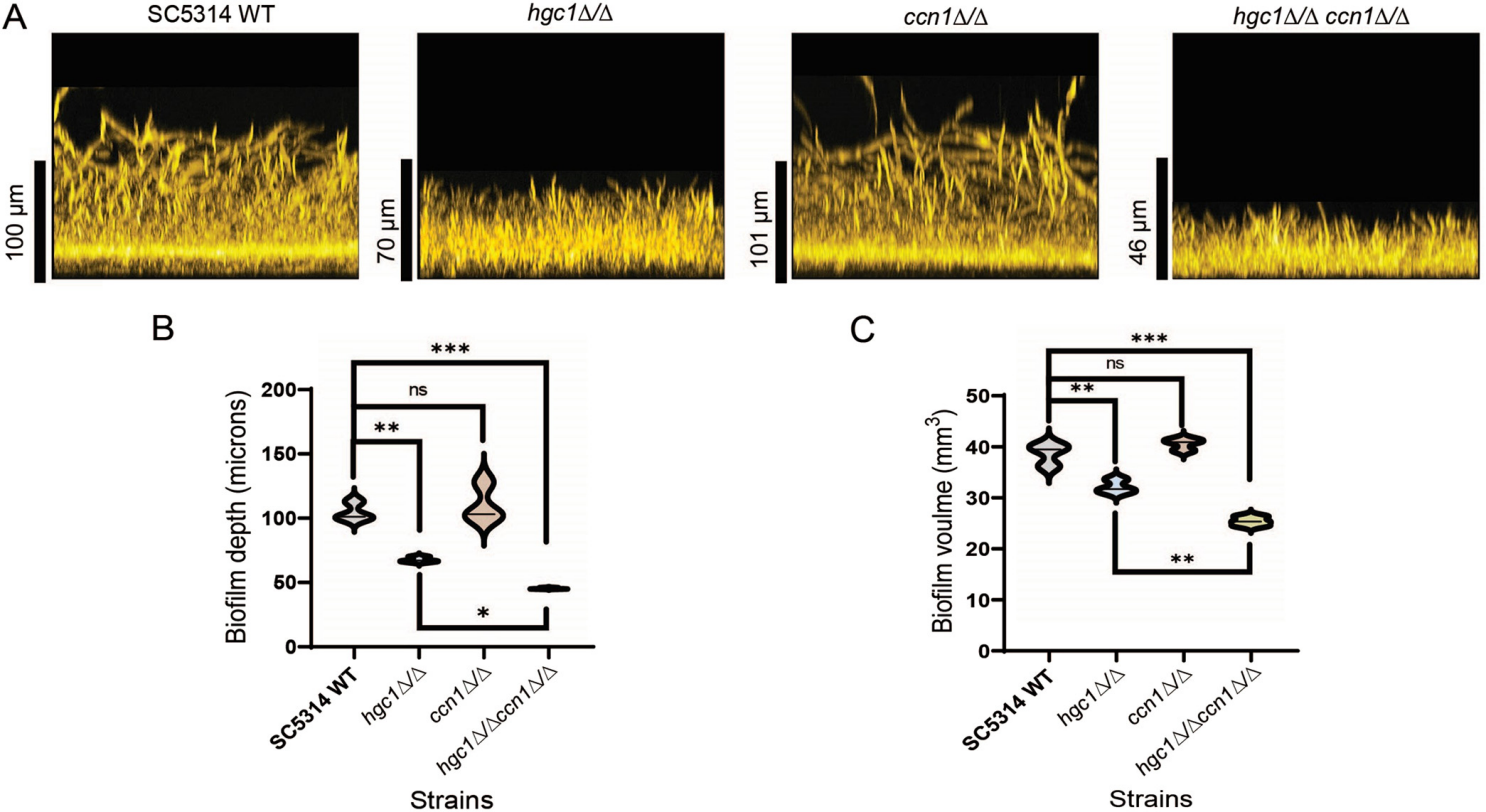
Genetic interaction between *HGC1* and *CCN1*. (A) Biofilm side view projections. Wild-type SC5314, *hgc1*Δ/Δ, *ccn1*Δ/Δ, and *hgc1*Δ/Δ *ccn1*Δ/Δ strains were assayed for biofilm formation in RPMI plus 10% serum at 37°C for 24 h (96-well plate). Fixed biofilms were stained using calcofluor white and imaged using a Keyence BZ-X800E fluorescence microscope. Representative side view images are shown, and the vertical scale bar represents 100 μm in length. (B and C) Violin plots showing the biofilm depth (in micrometers) and volume (in cubic millimeters) distributions of the indicated strains, respectively. Biofilm depth and volume were measured with the software ImageJ. The significant differences were calculated between the pair of means using Sidak’s multicomparison test: *, *P* < 0.05; **, *P* < 0.01; ***, *P* < 0.00; ns, *P* > 0.05.

The hypothesis that G1 cyclins have shared function under biofilm conditions predicts that deletion of G1 cyclin genes in the *hgc1*Δ/Δ background may increase the severity of its biofilm defect. *CLN3* is essential for C. albicans growth (14, 16, 34); *CCN1* is not. We constructed a homozygous *hgc1*Δ/Δ *ccn1*Δ/Δ mutant in the SC5314 background and compared single and double mutant biofilm growth in RPMI-plus-serum medium at 37°C for 24 h. The *ccn1*Δ/Δ mutant produced biofilm (110-μm depth and 41-mm^3^ volume) comparable to that of the wild-type strain (104-μm depth and 39-mm^3^ volume) (Fig. 7A, B, and C). However, biofilm growth of the *hgc1*Δ/Δ *ccn1*Δ/Δ mutant (45-μm depth and 25-mm^3^ volume) was significantly reduced compared to that of the *hgc1*Δ/Δ mutant (79-μm depth and 31-mm^3^ volume) (Fig. 7A, B, and C). *hgc1*Δ/Δ and *hgc1*Δ/Δ *ccn1*Δ/Δ mutants did not show significant differences in hyphal growth under these conditions (Fig. S3). Because deletion of the two cyclin genes causes a more severe defect than deletion of either gene alone, we conclude that *HGC1* and *CCN1* contribute independently to biofilm formation.

10.1128/mbio.03498-22.3 FIG S3 Hyphal growth during biofilm formation in SC5314 *hgc1*Δ/Δ and *hgc1*Δ/Δ *ccn1*Δ/Δ mutants. Apical view projections from the biofilms of the indicated strains were obtained using maximum-intensity Z-projection of ~20 planes at an 0.45-μm step size. Images were generated with a Keyence BZ-X800E fluorescence microscope. White scale bars in each panel are 100 μm in length. Download FIG S3, PDF file, 0.2 MB. Copyright © 2023 Sharma et al. 2023 Sharma et al. https://creativecommons.org/licenses/by/4.0/ This content is distributed under the terms of the Creative Commons Attribution 4.0 International license.

### Screen of cyclin genes for potential regulators of hyphal growth.

Our results argue that all three G1 cyclins contribute to hyphal growth under infection-relevant conditions. This prompted us to investigate the role of other cyclin genes in hyphal development. There are 6 other prospective cyclin genes in the C. albicans genome; they encode G2 cyclins (*CLB2* and *CLB4*) and *PHO85* cyclins (*CLG1* and *PCL1*, -*2*, -*5*, and -*7*). We tested possible improvement of biofilm formation by overexpression of each gene in the SC5314 wild-type and *hgc1*Δ/Δ strains. In each strain one allele of a cyclin gene was fused to the *RBT5* promoter (33). We assayed the strains for biofilm growth under conditions that magnify the *hgc1*Δ/Δ defect (RPMI medium for 24 h). SC5314 WT and its six cyclin-overexpression strains promoted biofilm growth of similar depth; however, overexpression of *PCL1* significantly increased volume compared to the WT (Fig. S4A, Fig. S5, and Fig. S6). Overexpression of 6 cyclin genes did not rescue the biofilm growth defect of the *hgc1*Δ/Δ mutant (Fig. S4B, C, and D), though increased expression of *PCL1* and *PCL7* in the *hgc1*Δ/Δ background increased the cell unit length (Fig. S4E and F). Therefore, these additional cyclin genes cannot rescue the *hgc1*Δ/Δ biofilm defect. These results indicate that the functional interaction of Hgc1, Ccn1, and Cln3 under biofilm conditions is specific for G1 cyclins.

## DISCUSSION

Biofilm growth of C. albicans requires hypha formation under virtually all circumstances (2, 9, 10). The G1 cyclin gene *HGC1* is well established as a central driver of hyphal development in C. albicans (7, 17). *HGC1* was shown to be required for biofilm formation by otherwise wild-type strains in one study (12), and it seemed very reasonable that the *hgc1*Δ/Δ mutation caused a biofilm defect due to its hyphal defect. Our results here indicate that *hgc1*Δ/Δ mutants produce hyphae during biofilm growth *in vitro*, under strong inducing conditions, and *in vivo*, in an oral infection model. Gene overexpression and double mutant analysis argues that two other G1 cyclin genes, *CCN1* and *CLN3*, can compensate partially for the absence of *HGC1* function.

### Planktonic cell Hgc1 function.

Prior studies revealed that some hyphal regulatory mutations have strain-dependent phenotypic impact (21). For example, *bcr1*Δ/Δ and *ume6*Δ/Δ mutations block hypha formation in some strains but not others (21). For *hgc1*Δ/Δ mutations, the severity of mutant phenotypes also varies among strains. In some cases, variation in the impact of *hgc1*Δ/Δ mutations reflects the strength of the corresponding wild-type phenotype. The clearest example is endothelial cell damage ability, where *hgc1*Δ/Δ mutations cause a significant defect only in high-damage strains. The extent of planktonic hyphal morphogenesis defects also followed this principle. In RPMI plus serum, *hgc1*Δ/Δ mutants of the strong hyphal formers SC5314, P76067, and GC75 yielded abundant pseudohyphae; *hgc1*Δ/Δ mutants of the weaker hyphal formers P57055 and 19F yielded few pseudohyphae and more abundant yeast and unclassifiable cells. Given that *hgc1*Δ/Δ mutants cause a planktonic hyphal defect in all strain backgrounds, our observations argue that Hgc1 is of uniformly critical importance for hypha formation under the planktonic growth conditions tested.

### Biofilm cell Hgc1 function.

We found two conditions under which *hgc1*Δ/Δ mutants produce hyphae. One condition was growth in the mouse oral infection model. Filaments were evident after 5 days of infection with *hgc1*Δ/Δ mutants of SC5314 and GC75. A second such condition was during biofilm growth *in vitro*. The *hgc1*Δ/Δ mutants of three strains produced biofilm with abundant long filaments. In this case, the filaments were sufficiently adherent to yield biofilms of considerable depth. These observations indicate that hypha formation can occur in the absence of Hgc1 under infection-relevant conditions.

How may biofilm growth conditions alter cyclin regulation of hypha formation? The high density of a biofilm community favors exchange of small molecules, including quorum-sensing signals and metabolic waste products (35). Sharing of extracellular vesicles also has a prominent impact on the biofilm phenotype (36). A simple hypothesis is that exchange of small molecules or vesicles rebalances or reconfigures cyclin activities, such that Ccn1 and Cln3 are more capable of assuming the function of Hgc1.

Polarized growth of dying *hgc1*Δ/Δ mutants was reported previously by Chen et al. (37). Arrest of DNA synthesis, achieved with hydroxyurea treatment, induced formation of polarized projections that resemble pseudohyphae by both wild-type and *hgc1*Δ/Δ mutant strains. This situation seems distinct from our observations here in that we found hyphae produced during growth, not during terminal cell cycle arrest. However, an interesting possibility is that the mechanism revealed by S phase arrest is utilized naturally under biofilm growth conditions, in keeping with the suggestion of Chen et al. (37).

## MATERIALS AND METHODS

### Media.

Strains were routinely grown on YPD (2% Bacto peptone, 2% dextrose, 1% yeast extract). Transformants were selected on YPD plus 400 μg/mL nourseothricin or complete synthetic medium (CSM) (2% dextrose, 1.7% Difco yeast nitrogen base with ammonium sulfate and auxotrophic supplements). For phenotypic assays, strains were grown in liquid RPMI 1640 medium (Sigma-Aldrich), adjusted to pH 7.4 and supplemented with 10% fetal bovine serum (Atlanta Biologicals), and Spider medium (nutrient broth, mannitol [Sigma M9647], K_2_HPO_4_, pH adjusted to 7.2 with NaOH).

### Strains.

C. albicans strains used in this study are listed in Table S1 in the supplemental material.

10.1128/mbio.03498-22.7 TABLE S1 Candida albicans strains used in this study and their genotypes. Download Table S1, PDF file, 0.2 MB. Copyright © 2023 Sharma et al. 2023 Sharma et al. https://creativecommons.org/licenses/by/4.0/ This content is distributed under the terms of the Creative Commons Attribution 4.0 International license.

### Primers and plasmid construction.

Plasmids and primers are listed in Tables S2 and S3, respectively. The full-length open reading frame (ORF) of the *HGC1* allele along with the promoter (1.3 kb) and terminator region (690 bp) was amplified from SC5314 genomic DNA using primers HGC1cloning F1 and HGC1cloning R1. The PCR product was then cloned in the pGEM-2T vector and sequenced to get the plasmid pGEM-*HGC1*.

10.1128/mbio.03498-22.8 TABLE S2 Plasmids used in this study and the relevant sources. Download Table S2, PDF file, 0.1 MB. Copyright © 2023 Sharma et al. 2023 Sharma et al. https://creativecommons.org/licenses/by/4.0/ This content is distributed under the terms of the Creative Commons Attribution 4.0 International license.

10.1128/mbio.03498-22.9 TABLE S3 List of oligonucleotide primers used in this study and their nucleotide sequences. Download Table S3, PDF file, 0.1 MB. Copyright © 2023 Sharma et al. 2023 Sharma et al. https://creativecommons.org/licenses/by/4.0/ This content is distributed under the terms of the Creative Commons Attribution 4.0 International license.

### Construction of *HGC1* deletion mutants.

Both alleles of *HGC1* were deleted in *his1*Δ/Δ derivatives of clinical isolates using the transient CRISPR-Cas9 system (38). Briefly, transformations included DNA cassettes (Cas9 DNA, HGC1 single guide RNA [sgRNA] DNA, NAT1-2 sgRNA DNA) and the *hgc1*Δ::*r1HIS1r1* repair template. The *HGC1* sgRNA DNA cassette was amplified using split-joint PCR with the primers sgRNA/F HGC1 and SNR52/R HGC1. The *hgc1*Δ::*r1HIS1r1* repair template was generated in two parts per reference 39, using plasmid pMH01 with primers HIS1 CRIME/F and HGC1 del rHIS1r KpnI/R, and plasmid pMH02 with primers HGC1 del rHIS1r SapI/F and HIS1 CRIME/R. Transformants were selected on CSM-His medium and replica plated onto YPD-plus-nourseothricin plates to screen for nourseothricin sensitivity. PCR genotyping was done with primers HGC1 check up/F and HGC1 check int/R for the absence of the *HGC1* ORF. Confirmation PCR with primers HGC1 check up/F and CdHIS1 Check Int/R verified integration of *HIS1* at the *hgc1*Δ locus.

Reconstituted strains had two copies of the SC5314 *HGC1* allele replacing the two *hgc1*Δ alleles via the concatemer assembly method (21). First, an *HGC1* cassette was amplified from plasmid pGEM-*HGC1* using primers HGC1cloning F1 and HGC1 3′R1-pNAT5′/R, containing concatenating homology to the *NAT1* marker. Then, the *NAT1* marker was amplified from pNAT using pNATF and pNAT 3′R-HGC1down/R1. The *HGC1*-containing cassette, corresponding *NAT1* marker, and r1 sgRNA DNA cassette were transformed into the *hgc1*Δ/Δ deletion mutant strains in all clinical isolate backgrounds, using a similar method as described earlier (37). Homozygosity at the *HGC1* locus was determined using the presence or absence of an *r1* scar (39, 40) and using PCR genotyping with the HGC1 check up/F and r1 check int/R primers.

### Construction of P*_RBT5_* cassettes of cyclin genes.

To generate heterozygous strains overexpressing cyclin genes *CCN1*, *CLN3*, *CLG1*, *PCL1*, *PCL2*, *PCL5*, *PCL7*, *CLB2*, and *CLB4*, a P*_RBT5_* cassette containing flanking homology to the gene upstream promoter region was amplified using primer sets CCN1OE F and CCN1OE R, CLN3OE F and CLN3OE R, CLG1OE F and CLG1OE R, PCL1OE F and PCL1OE R, PCL2OE F and PCL2OE R, PCL5OE F and PCL5OE R, PCL7OE F and PCL7OE R, CLB2OE F and CLB2OE R, and CLB4OE F and CLB4OE R, from plasmid pTH10 (33).

The sgRNA cassettes for the 5′ regions of each gene were generated using split-joint PCR using primers sgRNA/F CCN1P-1 and SNR52/R CCN1P-1, sgRNA/F CLN3P-1 and SNR52/R CLN3P-1, sgRNA/F CLG1P-1 and SNR52/R CLG1P-1, sgRNA/F PCL1P-1 and SNR52/R PCL1P-1, sgRNA/F PCL2P-1 and SNR52/R PCL2P-1, sgRNA/F PCL5P-1 and SNR52/R PCL5P-1, sgRNA/F PCL7P-1 and SNR52/R PCL7P-1, sgRNA/F CLB2P-1 and SNR52/R CLB2P-1, and sgRNA/F CLB4P-1 and SNR52/R CLB4P-1. The SC5314 WT and SC5314 *hgc1*Δ mutant strains were then transformed with 3 μg of the respective P*_RBT5_* cassette and 1 μg of sgRNA DNA cassette along with 1 μg of Cas9.

Transformants were selected on YPD-plus-nourseothricin plates for the resistant phenotype and were genotyped by PCR using primers CCN1 CHF and CCN1 CHR for the presence of one copy of the native *CCN1* promoter and CCN1 FCHF and NAT CHR for the presence of the P*_RBT5_* cassette in the target gene promoter region. Similarly, other gene constructs were genotyped using respective primer sets (Table S3).

### Construction of *CCN1* deletion mutants.

Both alleles of *CCN1* were deleted in the SC5314 wild type and its *hgc1*Δ/Δ mutant strain using a transient CRISPR-Cas9 system (38). Briefly, transformations included DNA cassettes (Cas9 DNA and CCN1-3 sgRNA DNA) and the *ccn1*Δ::*rNATr* repair template. The *CCN1-3* sgRNA DNA cassette was amplified using split-joint PCR with the primers sgRNA/F CCN1-3 and SNR52/R CCN1-3. The *ccn1*Δ::*rNATr* repair template was generated in two parts as per reference 39, using plasmid pMH05 with primers NAT CRIME/F and CCN1 del rNATrXmaI/R, and plasmid pMH06 with primers CCN1 del rNATrBamHI/F and NAT CRIME/R. Transformants were selected on YPD-plus-nourseothricin plates to screen for nourseothricin resistance. PCR genotyping was done with primers CCN1 CHF and CCN1 CHR for the absence of the *CCN1* ORF. Confirmation PCR with primers CCN1 CHF and NAT CHR verified integration of *NAT* at the *ccn1*Δ locus.

### Filamentation assays and imaging.

Hypha formation was assayed essentially as previously described (21). Calcofluor-stained cells were then imaged with a Zeiss fluorescence microscope.

### Biofilm growth and imaging.

To assay biofilm formation in a 96-well plate (Greiner 96-well plate; catalog no. 655090), strains were inoculated to an optical density at 600 nm (OD_600_) of 0.5 from overnight cultures into 100 μL of prewarmed RPMI with 10% fetal bovine serum (FBS) and RPMI. First, the cells were incubated in a shaker incubator at 37°C for 90 min with mild shaking (60 rpm) to allow adherence, and then each well was gently washed twice with phosphate-buffered saline (PBS). Next, 100 μL of prewarmed RPMI with 10% FBS or RPMI was added into each well, and cells were allowed to form biofilm in a shaker incubator with 60 rpm at 37°C for 24 h. At that time, the medium was discarded from each well, and biofilms were fixed by incubation with 100 μL of 4% formaldehyde in PBS solution for 1 h and then gently washed twice with PBS. Biofilms were stained with calcofluor white (200 μg/mL in PBS) overnight at room temperature (RT) with mild shaking (60 rpm), and then each well was gently washed twice with PBS. To clarify biofilms in the 96-well plates, we used 100% TDE (thiodiethanol), which has a refractive index of 1.521. We removed the PBS from each well and added 100 μL of 50% TDE in PBS. We incubated the 96-well plate at room temperature for an hour and then removed the solution from each well. Finally, 100 μL of 100% TDE solution was added to the wells and incubated at RT for an hour, when the clarified biofilm was transparent. The biofilms for *hgc1*Δ/Δ mutant strains (grown in RPMI with 10% FBS) were imaged using a Zeiss Axiovert 200 microscope with a Zeiss 25× 0.8-numerical-aperture (NA) multi-immersion objective. The biofilms for strains overexpressing cyclin genes and *CCN1* deletion were imaged using a Keyence BZ-X800E fluorescence microscope.

### Biofilm image processing.

Optical sections of the biofilms were collected in several series of planes at an 0.45- or 0.85-μm step size. The stacks were concatenated and processed using Fiji software (41). The images were processed using the Background Subtract plugin. The side view projection images were obtained by reslicing the stack and using the maximum intensity Z-projection. The scale of the stacked side view images was adjusted based on the objective used for the imaging. The biofilm depth measurements are obtained from the side view projection images. The biofilm volume was measured from the stacked biofilm image. The threshold value for the image was selected based on the intensity of the pixels to separate the pixels of interest from the background surface. The macro code runs through each image in the stack to sum the area measurements. It further multiplies this sum by the depth of each slice to calculate the volume. Overall, this analysis measures the total volume of the pixels per area parallel to the background surface that has an intensity greater than a specified threshold value.

### Endothelial cell damage assay.

The extent of the endothelial cell damage caused by the different strains of C. albicans was measured using the previously described ^51^Cr release assay (28, 42). The inoculum was 4 × 10^4^ organisms per well of endothelial cells. Each assay was performed in triplicate on three separate occasions.

### Biofilm quantification by XTT assay.

The quantification of biofilm growth was done using an XTT metabolic assay (43). Briefly, XTT (Sigma-Aldrich) was freshly prepared as a saturated solution in PBS (1 mg/mL). Before use, the menadione solution was prepared in acetone and added to the XTT to a final concentration of 1 μM. Strains were inoculated from overnight cultures to an OD_600_ of 1.67 into 1 mL of prewarmed RPMI with 10% serum. From this culture, 2 μL was inoculated into 100 μL prewarmed RPMI with 10% serum in a 96-well plate. Each strain was done in triplicate. The final OD_600_ of the culture was 0.033 (~10^6^ cells). The plate was incubated for 90 min at 37°C in a shaking incubator (60 rpm) and then washed with PBS. After that, 100 μL of prewarmed RPMI with 10% serum was added and then incubated at 37°C for 24 h in a shaking incubator. After washing with PBS, 100 μL of XTT-menadione solution was added and incubated at 37°C for 1 h. The solutions were transferred into a new 96-well plate, and OD_492_ was measured in each well using a plate reader.

### Mouse model of oropharyngeal candidiasis.

C. albicans strains were tested for virulence in the mouse model of oropharyngeal candidiasis (OPC) described previously (44). Briefly, male BALB/c mice were immunosuppressed with cortisone acetate (225 mg/kg of body weight; Sigma-Aldrich) administered every other day, starting at day −1 relative to infection. The mice were inoculated by placing a calcium alginate swab saturated with C. albicans blastospores sublingually for 75 min. Mice were sacrificed after 5 days of infection. The tongues were harvested, weighed, and cut in half. One half was weighed and homogenized for quantitative culture, and the other half was processed for histopathological analysis.

### Software.

Images were arranged or adjusted using Fiji software. All statistical analyses were carried out using GraphPad Prism, version 8.4.2.
