# Supplementary material for: Hgc1 Independence of Biofilm Hyphae in Candida albicans
Source: mBio. 2023 Feb 13;14(2):e03498-22. doi: 10.1128/mbio.03498-22 (PMC10128054; doi:10.1128/mbio.03498-22)
Supplement: FIG S1 [file mbio.03498-22-s0001.pdf]

Supplementary Figure S1

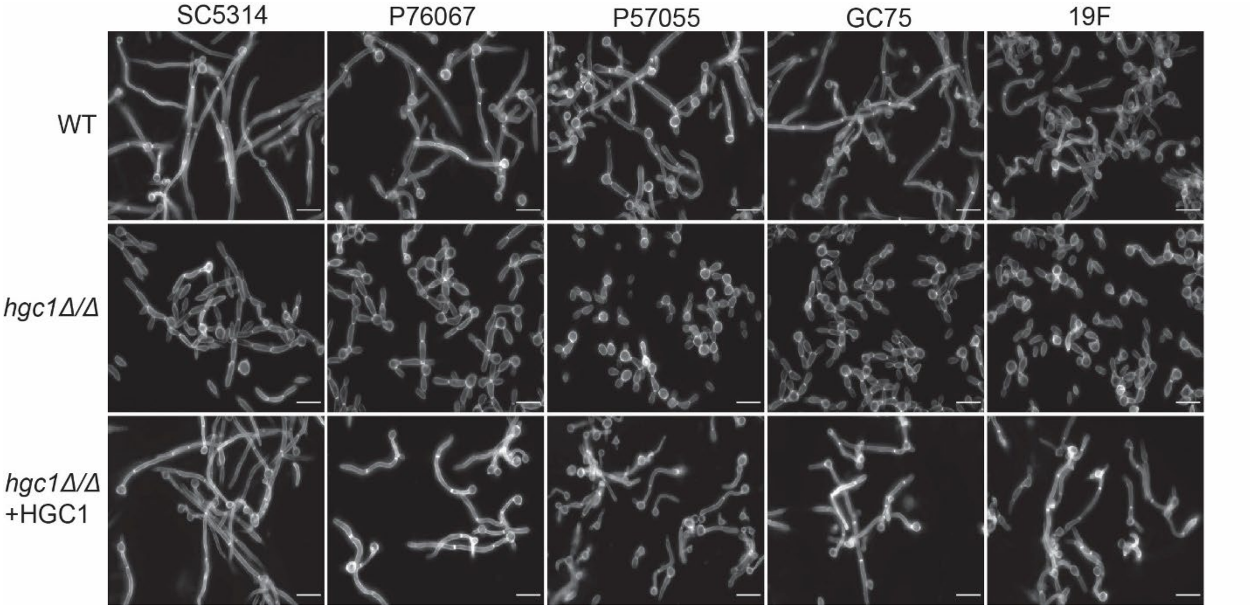

Fig. S1: Filamentation assay of reconstituted strains. The *HGC1* allele from SC5314 was reconstituted in the mutants of all clinical isolates. The reconstituted strains were grown in RPMI + 10% serum at 37°C for 4 hours alongside wild-type and *hgc1Δ/Δ* mutant strains in the corresponding clinical isolate backgrounds. Fixed cells were stained with Calcofluor-white and imaged using fluorescence microscopy. The white scale bars in each panel are 16 µm in length.
