# Supplementary material for: Hgc1 Independence of Biofilm Hyphae in Candida albicans
Source: mBio. 2023 Feb 13;14(2):e03498-22. doi: 10.1128/mbio.03498-22 (PMC10128054; doi:10.1128/mbio.03498-22)
Supplement: FIG S2 [file mbio.03498-22-s0002.pdf]

Supplementary Figure S2

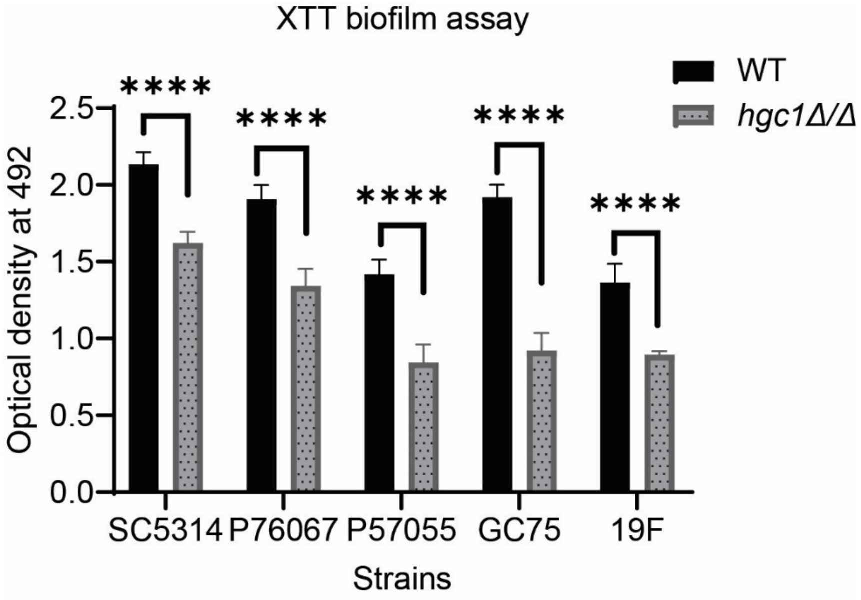

Fig. S2: XTT reduction assay. The effect of *hgc1Δ/Δ* mutation on the biofilm growth across five clinical isolates was measured in RPMI+ 10% serum using XTT assay. Error bars indicate standard deviations from 3 biological replicates assayed. Data were analyzed using Sidak's multicomparison test. (****, $p < 0.0001$).
