# Supplementary material for: Hgc1 Independence of Biofilm Hyphae in Candida albicans
Source: mBio. 2023 Feb 13;14(2):e03498-22. doi: 10.1128/mbio.03498-22 (PMC10128054; doi:10.1128/mbio.03498-22)
Supplement: FIG S4 [file mbio.03498-22-s0004.pdf]

Sharma et al. Supplementary information

Supplementary Figure S4

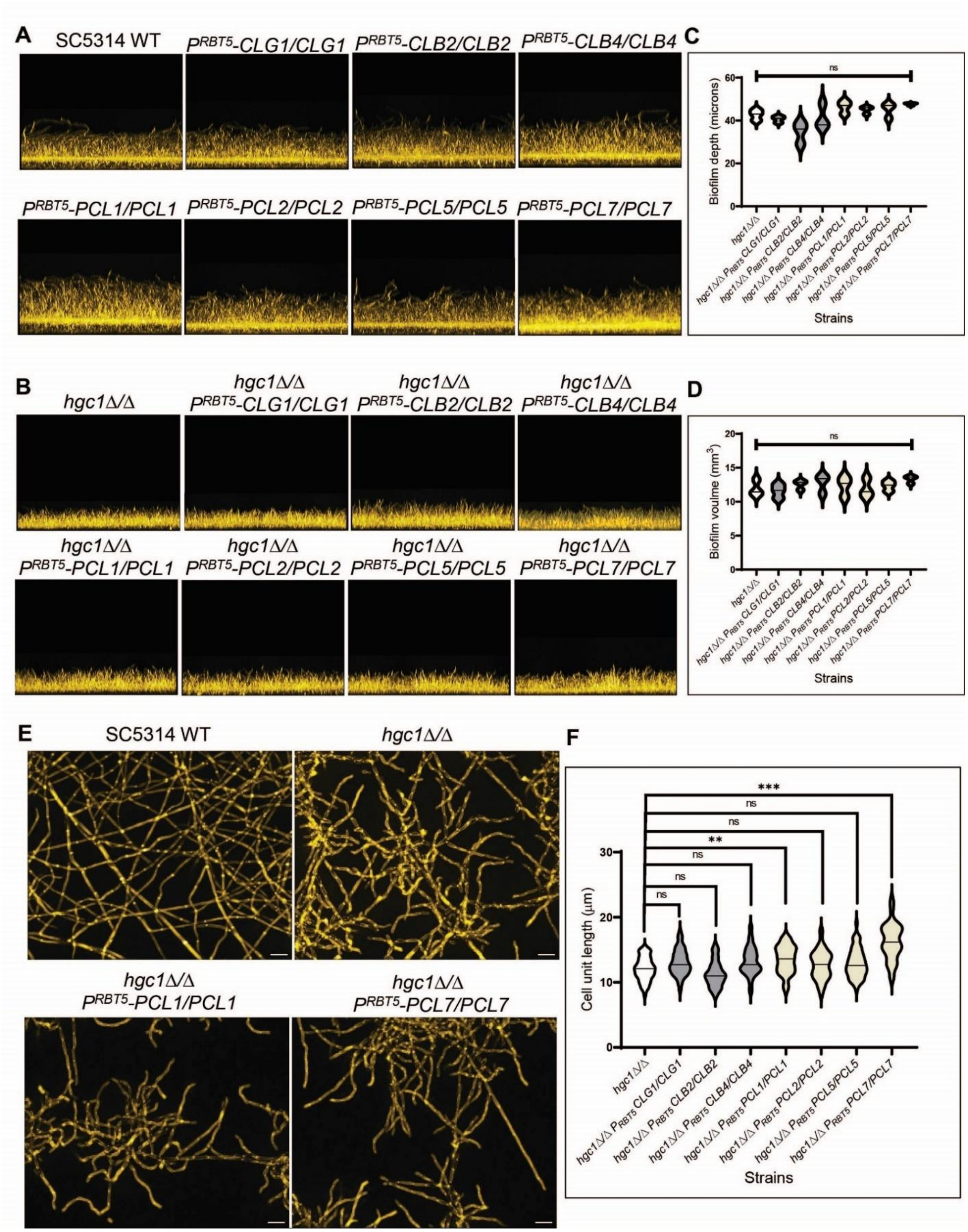

Fig. S4: Overexpression of cyclin genes *PCL1* and *PCL7* induces hyphal growth during biofilm formation in SC5314 *hgc1*Δ/Δ mutant. (A) and (B) Biofilm side view projections**.** Wild-type SC5314 and its derivative strains were assayed for biofilm formation under *in vitro* conditions. All strains were grown in RPMI at 37˚C for 24 hours (96-well plate). Fixed biofilms were stained using Calcofluor white and imaged using a Keyence BZ-X800E fluorescence microscope. Representative side view images are shown, and the vertical scale bar represents the 100 µm in length. **(C) and (D)** Violin plots showing the biofilm depth (in microns) and volume (in mm^3) distributions of the indicated strains respectively. Biofilm depth and volume were measured with the software ImageJ. The significant differences were calculated between the pair of means using Sidak's multicomparison test: ns, $p > 0.05$**. (E)** Apical view projections from the biofilms of the indicated strains were obtained using maximum intensity Z-projection of ~20 planes at 0.45 µm step-size. White scale bars in each panel are 100 µm in length. **(F)** Violin plots showing the distribution of cell unit lengths of the indicated strains. Cell unit lengths were quantified with ImageJ, with a minimum of five fields and 50 cells for each strain background. The significant differences were calculated between the pair of means using Sidak's multicomparison test: **, $p < 0.01$; ***, $p < 0.001$.
