# Supplementary material for: Hgc1 Independence of Biofilm Hyphae in Candida albicans
Source: mBio. 2023 Feb 13;14(2):e03498-22. doi: 10.1128/mbio.03498-22 (PMC10128054; doi:10.1128/mbio.03498-22)
Supplement: FIG S5 [file mbio.03498-22-s0005.pdf]

Sharma et al. Supplementary information

Supplementary Figure S5

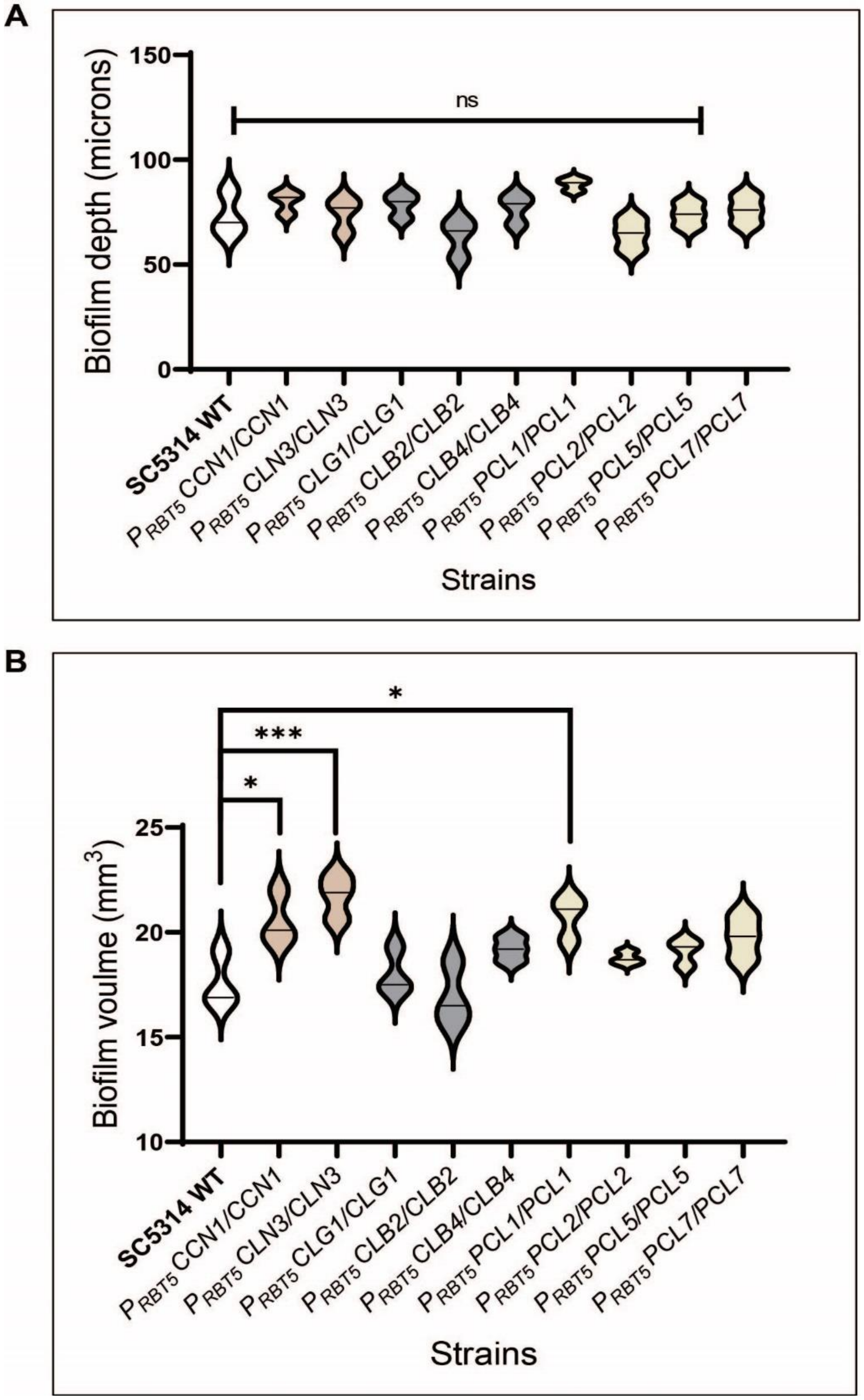

Fig. S5: Biofilm depth and volume of SC5314 wild type strains overexpressing cyclin genes. Biofilm depth (in micrometers) and volume (in cubic millimeters) of all the mentioned strains grown in RPMI medium at 37°C for 24 hours were measured with Image J. (**A) and (B)** Violin plots showing the biofilm depth (in microns) and volume (in mm^3) distributions of the indicated strains respectively. The significant differences were calculated between the pair of means using Sidak's multicomparison test: ns, $p > 0.05$: *, $p < 0.05$; ***, $p < 0.001$.
