# Supplementary material for: Hgc1 Independence of Biofilm Hyphae in Candida albicans
Source: mBio. 2023 Feb 13;14(2):e03498-22. doi: 10.1128/mbio.03498-22 (PMC10128054; doi:10.1128/mbio.03498-22)
Supplement: FIG S6 [file mbio.03498-22-s0006.pdf]

Supplementary Figure S6

	Biofilm depth		Biofilm volume	
**Strains**	**WT (µm)**	***hgc1*∆/∆ (µm)**	**WT (mm^3)**	***hgc1*∆/∆ (mm^3)**
SC5314	73	43	18	12
P_{RBT5} *CCN1/CCN1*	80	63	21	15
P_{RBT5} *CLN3/CLN3*	74	68	22	15
P_{RBT5} *CLB2/CLB2*	63	35	17	13
P_{RBT5} *CLB4/CLB4*	77	41	19	13
P_{RBT5} *CLG1CLG1*	79	41	18	12
P_{RBT5} *PCL1/PCL1*	88	46	21	12
P_{RBT5} *PCL2/PCL2*	65	45	19	12
P_{RBT5} *PCL5/PCL5*	74	45	19	12
P_{RBT5} *PCL7/PCL7*	76	48	20	13

Fig. S6: Biofilm depth and volume of strains overexpressing different cyclin genes. Biofilm depth (in micrometers) and volume (in cubic millimeters) of all the mentioned strains grown in RPMI medium at 37°C for 24 hours were measured with Image J. The values represent the means of the triplicate biofilms of each strain background.
