# Supplementary material for: Hgc1 Independence of Biofilm Hyphae in Candida albicans
Source: mBio. 2023 Feb 13;14(2):e03498-22. doi: 10.1128/mbio.03498-22 (PMC10128054; doi:10.1128/mbio.03498-22)
Supplement: FIG S3 [file mbio.03498-22-s0003.pdf]

Sharma et al. Supplementary information

Supplementary Figure S3

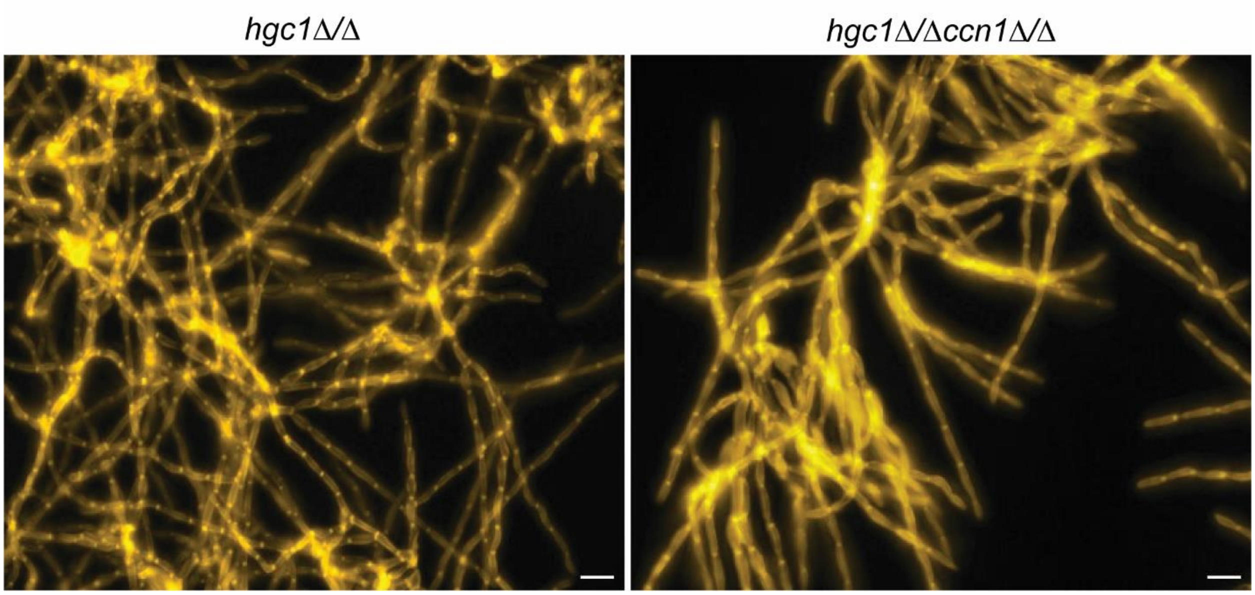

Fig. S3: Hyphal growth during biofilm formation in SC5314 *hgc1*Δ/Δ and *hgc1*Δ/Δ *ccn1*Δ/Δ mutants. Apical view projections from the biofilms of the indicated strains were obtained using maximum intensity Z-projection of ~20 planes at 0.45 μm step-size. Images were generated with a Keyence BZ-X800E fluorescence microscope. White scale bars in each panel are 100 μm in length.
