# Supplementary material for: Hgc1 Independence of Biofilm Hyphae in Candida albicans
Source: mBio. 2023 Feb 13;14(2):e03498-22. doi: 10.1128/mbio.03498-22 (PMC10128054; doi:10.1128/mbio.03498-22)
Supplement: TABLE S1 [file mbio.03498-22-s0007.pdf]

Supplementary Table S1

Strain no.	Strain	Species	Genotype/Parent
ASM1	SC5314	*C.albicans*	Wild type clinical isolate
ASM5	P76067	*C.albicans*	Wild type clinical isolate
ASM3	P57055	*C.albicans*	Wild type clinical isolate
ASM7	GC75	*C.albicans*	Wild type clinical isolate
ASM9	19F	*C.albicans*	Wild type clinical isolate
ASM11	SC5314 His-	*C.albicans*	*his1Δ::rNAT1r/his1Δ::rNAT1r*
ASM15	P76067 His-	*C.albicans*	*his1Δ::rNAT1r/his1Δ::rNAT1r*
ASM13	P57055 His-	*C.albicans*	*his1Δ::rNAT1r/his1Δ::rNAT1r*
ASM17	GC75 His-	*C.albicans*	*his1Δ::rNAT1r/his1Δ::rNAT1r*
ASM19	19F His-	*C.albicans*	*his1Δ::rNAT1r/his1Δ::rNAT1r*
ASM35	SC5314 *hgc1Δ/Δ*	*C.albicans*	*hgc1Δ::r1HIS1r1/hgc1Δ::r1HIS1r1 his1Δ::r3/his1Δ::r3*
ASM27	P76067 *hgc1Δ/Δ*	*C.albicans*	*hgc1Δ::r1HIS1r1/hgc1Δ::r1HIS1r1 his1Δ::r3/his1Δ::r3*
ASM33	P57055 *hgc1Δ/Δ*	*C.albicans*	*hgc1Δ::r1HIS1r1/hgc1Δ::r1HIS1r1 his1Δ::r3/his1Δ::r3*
ASM120	GC75 *hgc1Δ/Δ*	*C.albicans*	*hgc1Δ::r1HIS1r1/hgc1Δ::r1HIS1r1 his1Δ::r3/his1Δ::r3*
ASM118	19F *hgc1Δ/Δ*	*C.albicans*	*hgc1Δ::r1HIS1r1/hgc1Δ::r1HIS1r1 his1Δ::r3/his1Δ::r3*
ASM334	SC5314RC	*C.albicans*	*hgc1Δ::HGC1-SC5314 NAT1/hgc1Δ::HGC1-SC5314-NAT1 his1Δ::r3/his1Δ::r3*
ASM389	P76067RC	*C.albicans*	*hgc1Δ::HGC1-SC5314-NAT1/hgc1Δ::HGC1-SC5314-NAT1 his1Δ::r3/his1Δ::r3*
ASM315	P57055RC	*C.albicans*	*hgc1Δ::HGC1-SC5314-NAT1/hgc1Δ::HGC1-SC5314-NAT1 his1Δ::r3/his1Δ::r3*
ASM397	GC75RC	*C.albicans*	*hgc1Δ::HGC1-SC5314-NAT1/hgc1Δ::HGC1-SC5314-NAT1 his1Δ::r3/his1Δ::r3*
ASM393	19FRC	*C.albicans*	*hgc1Δ::HGC1-SC5314-NAT1/hgc1Δ::HGC1-SC5314-NAT1 his1Δ::r3/his1Δ::r3*
ASM524	SC5314 P_{RBT5}-*CCN1/CCN1*	*C.albicans*	*CCN1::*P_{RBT5}-*CCN1/CCN1*
ASM527	SC5314 *hgc1Δ/Δ* P_{RBT5}-*CCN1/CCN1*	*C.albicans*	*hgc1Δ::r1HIS1r1/hgc1Δ::r1HIS1r1 CCN1::*P_{RBT5}-*CCN1/CCN1 his1Δ::r3/his1Δ::r3*
ASM530	SC5314 P_{RBT5}-*CLN3/CLN3*	*C.albicans*	*CLN3::*P_{RBT5}-*CLN3/CLN3*
ASM533	SC5314 *hgc1Δ/Δ* P_{RBT5}-*CLN3/CLN3*	*C.albicans*	*hgc1Δ::r1HIS1r1/hgc1Δ::r1HIS1r1 CLN3::*P_{RBT5}-*CLN3/CLN3 his1Δ::r3/his1Δ::r3*
ASM545	SC5314 P_{RBT5}-*CLG1/CLG1*	*C.albicans*	*CLG1::*P_{RBT5}-*CLG1/CLG1*

ASM548	SC5314 *hgc1Δ/Δ* P_{RBT5}-*CLG1/CLG1*	*C.albicans*	*hgc1Δ::r1HIS1r1/hgc1Δ::r1HIS1r1 CLG1::*P_{RBT5}-*CLG1/CLG1 his1Δ::r3/his1Δ::r3*
ASM595	SC5314 P_{RBT5}-*CLB2/CLB2*	*C.albicans*	*CLB2::*P_{RBT5}-*CLB2/CLB2*
ASM597	SC5314 *hgc1Δ/Δ* P_{RBT5}-*CLB2/CLB2*	*C.albicans*	*hgc1Δ::r1HIS1r1/hgc1Δ::r1HIS1r1 CLB2::*P_{RBT5}-*CLB2/CLB2 his1Δ::r3/his1Δ::r3*
ASM553	SC5314 P_{RBT5}-*CLB4/CLB4*	*C.albicans*	*CLB4::*P_{RBT5}-*CLB4/CLB4*
ASM555	SC5314 *hgc1Δ/Δ* P_{RBT5}-*CLB4/CLB4*	*C.albicans*	*hgc1Δ::r1HIS1r1/hgc1Δ::r1HIS1r1 CLB4::*P_{RBT5}-*CLB4/CLB4 his1Δ::r3/his1Δ::r3*
ASM570	SC5314 P_{RBT5}-*PCL1/PCL1*	*C.albicans*	*PCL1::*P_{RBT5}-*PCL1/PCL1*
ASM574	SC5314 *hgc1Δ/Δ* P_{RBT5}-*PCL1/PCL1*	*C.albicans*	*hgc1Δ::r1HIS1r1/hgc1Δ::r1HIS1r1 PCL1::*P_{RBT5}-*PCL1/PCL1 his1Δ::r3/his1Δ::r3*
ASM577	SC5314 P_{RBT5}-*PCL2/PCL2*	*C.albicans*	*PCL2::*P_{RBT5}-*PCL2/PCL2*
ASM581	SC5314 *hgc1Δ/Δ* P_{RBT5}-*PCL2/PCL2*	*C.albicans*	*hgc1Δ::r1HIS1r1/hgc1Δ::r1HIS1r1 PCL2::*P_{RBT5}-*PCL2/PCL2 his1Δ::r3/his1Δ::r3*
ASM560	SC5314 P_{RBT5}-*PCL5/PCL5*	*C.albicans*	*PCL5::*P_{RBT5}-*PCL5/PCL5*
ASM565	SC5314 *hgc1Δ/Δ* P_{RBT5}-*PCL5/PCL5*	*C.albicans*	*hgc1Δ::r1HIS1r1/hgc1Δ::r1HIS1r1 PCL5::*P_{RBT5}-*PCL5/PCL5 his1Δ::r3/his1Δ::r3*
ASM586	SC5314 P_{RBT5}-*PCL7/PCL7*	*C.albicans*	*PCL7::*P_{RBT5}-*PCL7/PCL7*
ASM591	SC5314 *hgc1Δ/Δ* P_{RBT5}-*PCL7/PCL7*	*C.albicans*	*hgc1Δ::r1HIS1r1/hgc1Δ::r1HIS1r1 PCL7::*P_{RBT5}-*PCL7/PCL7 his1Δ::r3/his1Δ::r3*
ASM647	SC5314 *ccn1Δ/Δ*	*C.albicans*	*ccn1Δ::NAT1/ccn1Δ::NAT1*
ASM650	SC5314 *hgc1Δ/Δ ccn1Δ/Δ*	*C.albicans*	*ccn1Δ::NAT1/ccn1Δ::NAT1 hgc1Δ::r1HIS1r1/hgc1Δ::r1HIS1r1his1Δ::r3/his1Δ::r3*

Supplementary Table S1: *Candida albicans* strains used in this study
