# Supplementary material for: Hgc1 Independence of Biofilm Hyphae in Candida albicans
Source: mBio. 2023 Feb 13;14(2):e03498-22. doi: 10.1128/mbio.03498-22 (PMC10128054; doi:10.1128/mbio.03498-22)
Supplement: TABLE S2 [file mbio.03498-22-s0008.pdf]

Supplementary Table S2

**Plasmid name**	**Description**	**Marker**	**Reference**
pNAT	*NAT1* marker	ampR	Min *et al*., 2016
pV1093	CaCas9/sgRNA expression vector	ampR	Vyas *et al*., 2015
pMH01	pRS424 carrying *C.d.HIS1* from pSN52 at KpnI site	ampR	Huang and Mitchell, 2017
pMH02	pRS424 carrying *C.d.HIS1* from pSN52 at SapI site	ampR	Huang and Mitchell, 2017
pGEM-*HGC1*	pGEM-2T	ampR	This study
pTH10	P_{RBT5} cassette	ampR	Mao *et al.,*2022
pMH05	YEp24 carrying *NAT1* from pNAT at BamHI site	ampR	Huang *et al.,* 2019
pMH06	YEp24 carrying *NAT1* from pNAT at XmaI site	ampR	Huang *et al.,* 2019

Supplementary Table S2: Plasmids used in this study
