# Supplementary material for: Hgc1 Independence of Biofilm Hyphae in Candida albicans
Source: mBio. 2023 Feb 13;14(2):e03498-22. doi: 10.1128/mbio.03498-22 (PMC10128054; doi:10.1128/mbio.03498-22)
Supplement: TABLE S3 [file mbio.03498-22-s0009.pdf]

**Supplementary Table S3**

| **Primer name** | **Sequence** |
|---|---|
| **HGC1 del rHIS1rSapI/F** | **ATCAAAGCATCAAACCAATACCCAACACTTTAATATCTAGGGTTTCCATTCACATATACACATATAAACATATATTAATACTCGAGGTCGACGGTATCG** |
| **HGC1 del rHIS1rKpnI/R** | **AAAACATCATTAAAATTTCATATCATAATAACAACATCTTTCTCCATTCTCCATTCTCTACTTTATCTTTCTCTCTTTCTCCAATACGCAAACCGCC** |
| **HIS1 CRIME/F** | **GCGCAAGAAGCCTCAACT** |
| **HIS1 CRIME/R** | **GAGCTACAGGGCTTGACC** |
| **sgRNA/F HGC1** | **GGTATCGATACCGATAATGAGTTTTAGAGCTAGAAATAGCAAGTTAAA** |
| **SNR52/R HGC1** | **TCATTATCGGTATCGATACCCAAATTAAAAATAGTTTACGCAAGTC** |
| **HGC1 check up/F** | **CTTACATTTTAGACGACCAACGG** |
| **HGC1 check int/R** | **CACCCATTACTTTACTTGCAATCC** |
| **CdHIS1 Check Int/R** | **GGCGCAACAGATATATTGGTGCTCG** |
| **SNR52/F** | **AAGAAAGAAAGAAAACCAGGAGTGAA** |
| **sgRNA/R** | **ACAAATATTTAAACTCGGGACCTGG** |
| **SNR52/N** | **GCGGCCGCAAGTGATTAGACT** |
| **sgRNA/N** | **GCAGCTCAGTGATTAAGAGTAAAGATGG** |
| **CaCas9/for** | **ATCTCATTAGATTTGGAACTTGTGGGTT** |
| **CaCas9/rev** | **TTCGAGCGTCCCAAAACCTTCT** |
| **pNAT F** | **TTTCCCAGTCACGACGTT** |
| **r1 check int/R** | **CACTAAAGGGAACAAAAGCTGG** |
| **HGC1 cloning F1** | **GGGCAAATCAAATTTTACACAAAAAG** |
| **HGC1 cloning R1** | **CGAATAAAGGATACTTTCCAGTAGTGTATT** |

| | |
|---|---|
| **HGC13'R1-pNAT5'/R** | **CGACGTCGGGCCCAATTCGCCCTATAGTGAGTCGTATTACAA TTCACTGGCCGTCGTTTTACAACGTCGTGACTGGGAAACGAA TAAA GGATACTTTCCAGTAGTGTATT** |
| **pNAT3'R-HGC1down R1** | **TAATCTAATCGAAGGTAAAAAAACAATCTTGAGTTGACCATAA AGTAATTGCCATTGAGACCGAAGTAACAATTCAGGAGTGGAAT TG TGAGCGGATA** |
| **sgRNA/F r1** | **GGGATCCACTAGTTCTAGAGGTTTTAGAGCTAGAAATAGCAAG T TAAA** |
| **SNR52/R r1** | **CTCTAGAACTAGTGGATCCCCAAATTAAAAATAGTTTACGCAA GT C** |
| **NAT1 CHR** | **GTTCTGTATCTATAAGCAGTATCATCCAAAGTAGT** |
| **CCN1OE F** | **CGTTATTTTTTTTTGTCGTCTTAATCATTAGTAGTAGTGAGA GACATTTTTTTTGGTGTTGTAAAAAAAAAATACGCGAATCAAG CTTGCCTCGTCCCC** |
| **CCN1OE R** | **TCAAGTATTGGATGGTATGGTCGACGTTTAATATGATGTGGA GGACCATATTTGACTCTTTGTTGTTGTAAAGATGTCATCGTTG GGAGCTCTCCCAATG** |
| **CLN3OE F** | **ACAATTATCTTTCACCCTAACATTCACTCATTTCATAGGTTTCC AAATATTCAATCCGATTCCAGTTTGATACGTGTGAAATCAAGC TTGCCTCGTCCCC** |
| **CLN3OE R** | **TGTAACTTGAAATTACTTGCTTTGATCAGGGATTGCATCATCC TAACTTGATGGAAAGCATCAGGTGAATTAGGAAACATCGTTG GGAGCTCTCCCAATG** |
| **CLB2OE F** | **GTCAAGACTAAAAAAAAAAACAACAAACCAAAAGAATCACTA AACAAAACATTTACTAAAAGGGAGAGAGAGAGAAAAGAATCA AGCTTGCCTCGTCCCC** |
| **CLB2OE R** | **CTTATCGACTCTTGATGCTGTACTTTTGATCTAGTAAGTCTAA ACTCATTTTCATTATTAGTTTTAGTGACTTGTGGCATCGTTGG GAGCTCTCCCAATG** |
| **CLB4OE F** | **TAATACACTGATCAAACGACAGTACTAAACTTACGAAAGCTTT CAGTTTCTACCGTGCTTCACAGCTTGTTCCTTTAGACATCAAG CTTGCCTCGTCCCC** |
| **CLB4OE R** | **AAATTGGCAATACTTTTGGCTCTAAGTCTTTGTTTTGTCAACT CATTTTCATCCGTTATGGATGATTTATAAGATCGCATCGTTGG GAGCTCTCCCAATG** |
| **PCL1OE F** | **CAAAATCTATTTTAATAACCAAAGACACGCCAAATTACTTTCT TTCTCTCTGTCTCTCTGTCTCCTTTCTTTCTTTCTTTATCAAGC TTGCCTCGTCCCC** |

| | |
|---|---|
| **PCL1OE R** | **ACAATCTTATGGATCATATCATGAGTGATTGGACCATTAAGAAATACTTTTAAAGCTTGTAAATCAATTGAAGAAACCATCGTTGGGAGCTCTCCCAATG** |
| **PCL2OE F** | **TTTTGAAGCAAAGCCAACAAAGTAAAACAATCGAAATTTGCTATCGTCAACAAAGAAAACAAAAAAAAAGGCTTATTGTGATCAAGCTTGCCTCGTCCCC** |
| **PCL2OE R** | **GTGGTTGCAACTAGAAAGTTGACCATATCTTGTGATACTGGCTGTCTTGAGAATATTTTCAAAGCTTCTTTGTCTGACATCGTTGGGAGCTCTCCCAATG** |
| **PCL5OE F** | **ATAGTATTATTTGGTGGCAAATGTCCTACTCCACAAACTGGTTTGTGATCTGTCCCGGTCAATGATGCAATCTCGTGTGAATCAAGCTTGCCTCGTCCCC** |
| **PCL5OE R** | **GTTTCTGTGGTGGTTGATTCCTTTGAAAAGGATGGTACACTTGTTTCTTTATAGGAAATAGGAGAAGTTGGAGATTGCATCGTTGGGAGCTCTCCCAATG** |
| **PCL7OE F** | **TTCCAACAAAGTTTTTTTTATTTTTTACTTTTTTTTACTATTTTTTGTTGTTGTTGATTGACTAATTGAATGTCTTCAAATCAAGCTTGCCTCGTCCCC** |
| **PCL7OE R** | **TGGTCTATGACCAGACCCCCTTCTCGATTGTTTGGGTCTATAGTGGTCGAGTTGGTGTCACCGTTATACTGTGATGTCATCGTTGGGAGCTCTCCCAATG** |
| **CLG1OE F** | **CCTATTCTCATCAAATATCTAAACTGAAATCACATTTCTTTCCTTCCTTCCTTCAAATTAACTTAATAAAGACTGTATATATCAAGCTTGCCTCGTCCCC** |
| **CLG1OE R** | **TGTTTAAGAATTCCGTGGCAAGCCCATCCAATAAAGGAGGGCATGTCACTTATCTGGTACTCCTGATACTCCTTATCCATCGTTGGGAGCTCTCCCAATG** |
| **CCN1 CHF** | **ACAAACCTTCACTTCTTCTTCTCCTCC** |
| **CCN1 CHR** | **CACAAAGAAACCTAGCAACAGCAGTTA** |
| **CCN1 FCHF** | **GTTGTTGCTGTTGTTGTTGTTGTGC** |
| **CLN3 CHF** | **TTTCTTCCATCAACACTAATCTGGTGC** |
| **CLN3 CHR** | **GCATTGACGACCCATTCTAATGAT** |
| **CLN3 FCHF** | **TCATCACTACCAAAGAACCACCTGATC** |
| **CLB2 CHF** | **AATAGGTGAGAGCAACCACAAGAAGAG** |
| **CLB2 CHR** | **GATGTTACAGAGACTTGAGGTGGTGAT** |
| **CLB2 FCHF** | **CATCAATCACCACCACCACCAC** |

| | |
|---|---|
| **CLB4 CHF** | **TAAGCAACCAAACGCTTCAAAGAGAG** |
| **CLB4 CHR** | **GCTGTTTCTGTGATGATTGGTGTTTGT** |
| **CLB4 FCHF** | **TAACAGTTTGCTTTACCTACCACGAG** |
| **PCL1 CHF** | **TTCGAGTTCTCCAGAGTCTCC** |
| **PCL1 CHR** | **GAAATGGATACAGCAACGGCAG** |
| **PCL1 FCHF** | **AATTAAAGAGAAACGCGACACC** |
| **PCL2 CHF** | **GTGTGTTGGCGTTGTTGTTGTTG** |
| **PCL2 CHR** | **GCTGGTTTCTCTTCAGTGGTTTGAC** |
| **PCL2 FCHF** | **GTCGTGACTGTCGTTTGATTGTATAAGG** |
| **PCL5 CHF** | **GTGGCAGCAGCAACAAATTGTG** |
| **PCL5 CHR** | **GTTTCTGTGGTGGTTGATTCCTTTGA** |
| **PCL5 FCHF** | **TTTGCCAGGAAAGTAGGTGCTC** |
| **PCL7 CHF** | **TGATACAAGAGACATTGCCAACATACA** |
| **PCL7 CHR** | **GACGCTGGATAAGAGTGATGTGATG** |
| **PCL7 FCHF** | **TGAACTCTAGTCAACCCACCAC** |
| **CLG1 CHF** | **CCCTGTGGTACACGACCTATCC** |
| **CLG1 CHR** | **GTGGCAAGCCCATCCAATAAAGG** |
| **CLG1 FCHF** | **ATTAAACTCTATCCAGCCCACCATTG** |
| **sgRNA/F CCN1P-1** | **TTTCAAAATTTTTTTATATTGTTTTAGAGCTAGAAATAGCAAG TTAAA** |
| **SNR52/R CCN1P-1** | **AATATAAAAAAATTTTGAAACAAATTAAAAATAGTTTACGCAA GTC** |
| **sgRNA/F CLN3P-1** | **AAATTGAATATTTTAAAAGAGTTTTAGAGCTAGAAATAGCAAG TTAAA** |
| **SNR52/R CLN3P-1** | **TCTTTTAAAATATTCAATTTCAAATTAAAAATAGTTTACGCAA GTC** |
| **sgRNA/F CLB2P-1** | **CTCATCGAACAAGATCTTACGTTTTAGAGCTAGAAATAGCAAG TTAAA** |

| | |
|---|---|
| **SNR52/R CLB2P-1** | **GTAAGATCTTGTTCGATGAGCAAATTAAAAATAGTTTACGCAAGTC** |
| **sgRNA/F CLB4P-1** | **AAGAAAAAAAGTTCTATATAGTTTTAGAGCTAGAAATAGCAAGTTAAA** |
| **SNR52/R CLB4P-1** | **TATATAGAACTTTTTTTCTTCAAATTAAAAATAGTTTACGCAAGTC** |
| **sgRNA/F PCL1P-1** | **GAGAACTCGAAATCAAAAAAGTTTTAGAGCTAGAAATAGCAAGTTAAA** |
| **SNR52/R PCL1P-1** | **TTTTTTGATTTCGAGTTCTCCAAATTAAAAATAGTTTACGCAAGTC** |
| **sgRNA/F PCL2P-1** | **GTGATGTTTGTTGACATTGCGTTTTAGAGCTAGAAATAGCAAGTTAAA** |
| **SNR52/R PCL2P-1** | **GCAATGTCAACAAACATCACCCAAATTAAAAATAGTTTACGCAAGTC** |
| **sgRNA/F PCL5P-1** | **AGCAGTGACAACTTATCTGCGTTTTAGAGCTAGAAATAGCAAGTTAAA** |
| **SNR52/R PCL5P-1** | **GCAGATAAGTTGTCACTGCTCAAATTAAAAATAGTTTACGCAAGTC** |
| **sgRNA/F PCL7P-1** | **AAAAGAAAAGAAAGCACATTGTTTTAGAGCTAGAAATAGCAAGTTAAA** |
| **SNR52/R PCL7P-1** | **AATGTGCTTTCTTTTCTTTTCAAATTAAAAATAGTTTACGCAAGTC** |
| **sgRNA/F CLG1P-1** | **GCTGACTCAGTTTGTAAGACGTTTTAGAGCTAGAAATAGCAAGTTAAA** |
| **SNR52/R CLG1P-1** | **GTCTTACAAACTGAGTCAGCCAAATTAAAAATAGTTTACGCAAGTC** |
| **NAT CRIME/F** | **CAGACGCGTTGAATTGTCC** |
| **NAT CRIME/R** | **CACCATGACCTCTATGTTCTGG** |
| **CCN1 del rNATrBamHI/F** | **CAATTTCCATTTTTGCATATATTAATTACTATTACTCAGTCCATTCGCTTTAAATCTATACATATATCCTAAATCTTATCGCTTTAATGCGGTAGTTTATCACAG** |
| **CCN1 del rNATrXmaI/R** | **TTTCTGATTTATATTAACGTCAACGTCAATATAAATTAAACAGGACAAAATGTATAGCTAGAAATAACACCATCATAACTGTGTGGTCGCCATGATCG** |
| **sgRNA/F CCN1-3** | **TAGTTGGTTGTACAGCATTAGTTTTAGAGCTAGAAATAGCAAGTTAAA** |

| | |
|---|---|
| **SNR52/R CCN1-3** | **TAATGCTGTACAACCAACTACAAATTAAAAATAGTTTACGCAA GTC** |

**Supplementary Table S3: List of primers and their nucleotide sequence.**
